# Genomics and transcriptomics landscapes associated to changes in insulin sensitivity in response to endurance exercise training

**DOI:** 10.1038/s41598-021-98792-1

**Published:** 2021-12-02

**Authors:** Louise Y. Takeshita, Peter K. Davidsen, John M. Herbert, Philipp Antczak, Matthijs K. C. Hesselink, Patrick Schrauwen, S. John Weisnagel, Jeremy M. Robbins, Robert E. Gerszten, Sujoy Ghosh, Mark A. Sarzynski, Claude Bouchard, Francesco Falciani

**Affiliations:** 1 Institute of Systems, Molecular and Integrative Biology, University of Liverpool, Crown Street, Liverpool, L69 7ZB UK; 2 Department of Nutrition and Movement Sciences, NUTRIM School for Nutrition and Translational Research in Metabolism, Maastricht University Centre, Maastricht, The Netherlands; 3 Diabetes Research Unit, Endocrinology and Nephrology Axis, CRCHU de Québec, Université Laval, Québec City, Canada; 4 Division of Cardiovascular Medicine, and Cardiovascular Research Center, Beth Israel Deaconess Medical Center, Boston, MA 02215 USA; 5 Centre for Computational Biology and Program in Cardiovascular and Metabolic Disorders, Duke-NUS Medical School, Singapore, Singapore; 6 Department of Exercise Science, Arnold School of Public Health, University of South Carolina, Columbia, SC USA; 7 Human Genomics Laboratory, Pennington Biomedical Research Center, Baton Rouge, LA USA; 8 Center for Molecular Medicine Cologne, University Hospital Cologne, 50931 Cologne, Germany

**Keywords:** Bioinformatics, Genetics, Physiology, Gene expression profiling

## Abstract

Despite good adherence to supervised endurance exercise training (EET), some individuals experience no or little improvement in peripheral insulin sensitivity. The genetic and molecular mechanisms underlying this phenomenon are currently not understood. By investigating genome-wide variants associated with baseline and exercise-induced changes (∆) in insulin sensitivity index (S_i_) in healthy volunteers, we have identified novel candidate genes whose mouse knockouts phenotypes were consistent with a causative effect on S_i_. An integrative analysis of functional genomic and transcriptomic profiles suggests genetic variants have an aggregate effect on baseline S_i_ and ∆S_i_, focused around cholinergic signalling, including downstream calcium and chemokine signalling. The identification of calcium regulated MEF2A transcription factor as the most statistically significant candidate driving the transcriptional signature associated to ∆S_i_ further strengthens the relevance of calcium signalling in EET mediated S_i_ response.

## Introduction

Regular endurance exercise is a strong physiological stimulus that plays an important role in skeletal muscle homeostasis. It leads to a multitude of functional improvements when performed regularly (i.e. exercise training) and is considered a cornerstone in the prevention of type 2 diabetes^1,2^ by increasing tissue responsiveness to circulating insulin. Skeletal muscle contraction and peripheral insulin action are highly inter-twined^3^. In fact, up to 80% of the in vivo insulin-mediated glucose disposal in the postprandial state occurs in skeletal muscle^4^, making it quantitatively the most important tissue for systemic glucose disposal. However, we and others have demonstrated that healthy individuals are highly heterogeneous in their ability to improve peripheral insulin sensitivity (S_i_) in response to endurance exercise training (EET)^5–7^. Notably, despite good adherence to the EET program, a significant percentage of individuals (up to ~ 20%) show no changes in S_i_ and some even demonstrate decreases in S_i_ values^5–7^. Furthermore, we have previously shown that such phenomenon is likely to include a substantial genetic component^8^ and that healthy individuals with high and low S_i_ responses to EET have different skeletal muscle gene expression patterns at baseline^6^.

A number of studies have been performed to understand the molecular basis of insulin resistance (IR), a pathological alteration in insulin sensitivity linked to many metabolic disorders, such as type 2 diabetes. Although the exact underlying cause of IR has not been fully elucidated, a number of major mechanisms, including oxidative stress, inflammation, insulin receptor mutations, endoplasmic reticulum stress, and mitochondrial dysfunction have been suggested^9^. Overall, however, the molecular mechanisms underlying variation in S_i_ in a healthy population and the heterogeneous ability to improve S_i_ through EET are currently not well understood.

Here we address this important question by computational analysis of genome-wide association study (GWAS) and skeletal muscle gene expression datasets derived from the HERITAGE Family Study. Our analysis identified several candidate genes linked to mechanisms of baseline S_i_, as well as training-induced changes in S_i_ (ΔS_i_). Homozygous mouse knockouts of four of these candidates show alterations in glucose disposal and other relevant phenotypes, suggesting that our approach is likely to have identified genes causally linked to S_i_. Furthermore, analysis of both GWAS and skeletal muscle transcriptomics data shows that a molecular signature linked to calcium-regulated cholinergic signalling may be an important component of the observed variation in S_i_ in a healthy population and predicts exercise-induced changes in S_i_ both in HERITAGE and an independent clinical exercise study.

## Methods

### HERITAGE family study

The sample, study design, and EET protocol of the HERITAGE study have been described elsewhere^10^. Briefly, for the Caucasian sample of HERITAGE, 479 sedentary adults (233 males) from 99 nuclear families composed of parents (≤ 65 years old) and offspring (≥ 17 years old) were defined as completers (> 95% of all exercise session requirements) following exposure to a standardized and fully monitored progressive 20-week EET program (frequency of cycle ergometer sessions was three times per week). Participants were all sedentary, but healthy at baseline and not taking medications for hypertension, diabetes, or dyslipidemia. A detailed description of the study design and methodology (including a table with demographic data) can be found in the Supplementary Information section.

### Intravenous glucose tolerance test (IVGTT) protocol

A frequently-sampled IVGTT was performed after an overnight fast (12 h), at baseline and post-intervention (24–36 h after the last exercise bout) following the protocol described in^11^. In premenopausal women, the test was scheduled to coincide with the follicular phase of the menstrual cycle. The S_i_ index (mU/[L × min]), which measures the ability of an increment in plasma insulin to enhance the net disappearance of glucose from plasma was derived using the MINMOD Millennium software^12^. Changes in S_i_ (∆S_i_) were calculated as post-training S_i_ minus baseline S_i_.

### GWAS genotype data processing

Single nucleotide polymorphism (SNP) genotyping (~ 325,000 SNPs, Illumina Human CNV370-Quad v3.0 BeadChips) on genomic DNA from lymphoblastoid cells was performed and subjected to extensive quality control as previously described^13^. SNPs excluded from association analyses were filtered according to the following criteria: (a) minor allele frequency < 5%, (b) violated Hardy–Weinberg equilibrium (p < 1 × 10^−6^), and (c) missing values in > 10% of individuals. SNPs are based on dbSNP build 151 with genomic coordinates for GRCh38 (hg38) assembly. To estimate linkage disequilibrium (LD), r^2^ correlation values between SNPs were calculated using default parameters in PLINK v1.9 (www.cog-genomics.org/plink/1.9/).

### GWAS analysis

Baseline S_i_ was adjusted for sex, age, log-transformed BMI, and weight-adjusted VO_2_max and ∆S_i_ was adjusted for log-transformed baseline S_i_, sex, age, log-transformed BMI, and weight-adjusted VO_2_max. Associations between the normalized trait residuals and SNP genotypes were investigated using additive linear mixed effect (LME) models that accounted for within family correlations (function lme of the ‘nlme’ R package v3.1^14^). Significance thresholds were calculated using the *SimpleM* method^15^ implemented in R programming language^16^. Conventional Bonferroni correction is overly conservative in genome-wide analyses due to high LD observed in genetic variants. The *SimpleM* method uses principal component analysis to calculate the effective number of independent tests, which resulted in 199,278 tests. A Bonferroni adjustment on this number results in a significance threshold of p < 2.51 × 10^−7^ (0.05/199,278). A suggestive significance threshold was set at p < 1 × 10^−5^. All statistical analyses were performed using R version 3.5.1. SNPs were mapped to genes based either on their position (located within a 20 kb window upstream and downstream of the gene) or if they have been identified as eQTL of a gene expressed in skeletal muscle tissue. Positional mapping was performed using MAGMA v1.07b and eQTL associations were retrieved from GTEx Portal release V8^17^.

### Candidate genes validation

We assessed the potential relevance of the candidate genes identified by the GWAS analysis by using a publicly available dataset from the International Mouse Phenotyping Consortium (IMPC) database^18^. We selected a panel of physiological measurements of relevance to S_i_. Details of the protocols are available from the database web site (https://www.mousephenotype.org/). Briefly, the ability to metabolize glucose has been assessed using three parameters derived from an intra-peritoneal glucose tolerance test (IPGTT). These were: (1) initial response to glucose challenge, (2) fasting blood glucose concentration and (3) the area of glucose response under the curve. Body composition was assessed by DEXA scan. Further characterization included a panel of blood measurements including insulin, cholesterol, glucose, glycerol, free fatty acids, and creatinine. In addition, respiratory exchange ratio was also available.

### Functional GWAS

To test whether genes within specific biological pathways are enriched by genetic associations with lower p-values than expected by chance, we applied GLOSSI^19^ from *cpvSNP* R package (v 1.18.0)^20^. First, GWAS results were pruned to keep only independent SNPs (r^2^ > 0.8) resulting in 249,035 SNPs. After positionally mapping the remaining SNPs to genes (± 20 kb window), their p-values were used as input to compute the estimate of enrichment within a given biological pathway. Resulting p-values were corrected for multiple testing using Bonferroni correction. Gene set collections used KEGG pathways (c2.cp.kegg.v7.1.entrez) retrieved from MSigDB v7.1^21,22^ and manually curated functional modules representing genes required for normal skeletal muscle activity^23^. Threshold for significantly enriched biological pathways was defined as p_adj_ < 0.05. In order to further investigate the most important pathways, we selected the most significant SNPs associations (alpha < 0.05) within the pathways identified by GLOSSI and remapped these on KEGG pathways using the web-based tool DAVID (version 6.8). Threshold for significant biological pathways was defined as p_adj_ < 0.05, provided in DAVID with Benjamini–Hochberg adjustment.

### RNA extraction and global gene expression profiling

Vastus lateralis muscle biopsies were also obtained in a subsample of the SNP-genotyped participants (n = 41) before and after (~ 96 h after final exercise session) the intervention using a percutaneous needle. Each biopsy was immediately frozen in liquid nitrogen and stored at – 80 °C until RNA preparation. RNA extraction as well as reverse transcription were done as previously described^24^. Affymetrix U133 + 2 arrays were used to quantitate global mRNA expression levels. The raw microarray CEL files are deposited in the public Gene Expression Omnibus (GEO) database^25^ under accession number GSE117070.

### Microarray data processing

Raw CEL files were Robust Multichip Average (RMA) normalized following removal of probes termed ‘absent’ in more than 80% of the samples by the MAS5 algorithm inside the ‘affy’ package (26,151 probesets discarded)^26^. Quality control plots of the polyA-control RNAs (spike-ins added right after RNA purification) highlighted a batch issue that was resolved by applying the COMBAT software^27^. The JetSet package was used to select a single ‘optimal’ probeset to represent each gene based on specificity, robustness against mRNA degradation, and MAS5 present call rate^28^. As the most representative probeset for each gene is selected, they have high splice isoform coverage.

### Gene set enrichment analysis (GSEA)

The entire skeletal muscle transcriptome was ranked by individually regressing pre-training mRNA expression levels against baseline S_i_ and ∆S_i_, using linear mixed effect models that accounted for within family correlations. Both outcome variables were adjusted by age, sex, log-transformed BMI, weight-adjusted VO_2_max, and type I fibre percentage (see^29^ for details on the fibre typing), with ∆S_i_ also being adjusted for baseline S_i_. Based on this ranking (Student *t*-statistic), we performed a pre-ranked GSEA using the default parameters in *clusterProfiler* v3.14.3 R package^30^ to identify candidate biological pathways significantly enriched in genes that are associated with baseline S_i_ and ∆S_i_ (either top or bottom of the distribution). Gene set collections used were transcription factor targets and KEGG biological pathways (c3.tft.v7.1.entrez.gmt and c2.cp.kegg.v7.1.entrez, respectively) retrieved from MSigDB v7.1^21^ and manually curated functional modules representing genes required for normal skeletal muscle activity^23^. Threshold for significant sets was defined as false discovery rate (FDR) < 0.05.

### Transcriptomics-based model to predict ∆S_i_

To test if baseline expression of MEF2A target genes is predictive of exercise-induced ∆S_i_ we applied a regression-based modelling approach allowing for pairwise interactions (function *lm* of the ‘stats’ R package^16^) between baseline expression of three genes. Only genes translating to proteins that interact with MEF2A were included and consisted of a total of 50 experimentally validated interactors with high confidence score (> 0.8) identified in the STRING database^31^ (Supplementary Table S1). All possible linear regression models based on all possible combinations of three-genes sets were examined (a total of 19,600 models). More precisely, we define:

$$\Delta S_{i} = a\theta_{1} + b\theta_{2} + c\theta_{3} + d\theta_{1} \theta_{2} + e\theta_{1} \theta_{3} + f\theta_{2} \theta_{3} + sex + VO2_{max/kg } + \varepsilon$$

where mRNA abundance is represented by θ and the noise model component by ε. Weight-adjusted VO2max and sex were included as covariates.

### Ethics declaration

The study protocol was approved by the Institutional Review Boards at each of the five participating centers of the HERITAGE Family Study consortium (Indiana University, Laval University, University of Minnesota, Texas A&M University, and Washington University at St. Louis). Written informed consent was obtained from each study participant. The subjects who were under 18 years, one of the parents gave consent in addition to the participant. This was an easy procedure to follow as we were recruiting whole nuclear families. All research was performed in accordance with the Declaration of Helsinki.

## Results

### Overview of the analysis strategy

**Figure 1:**
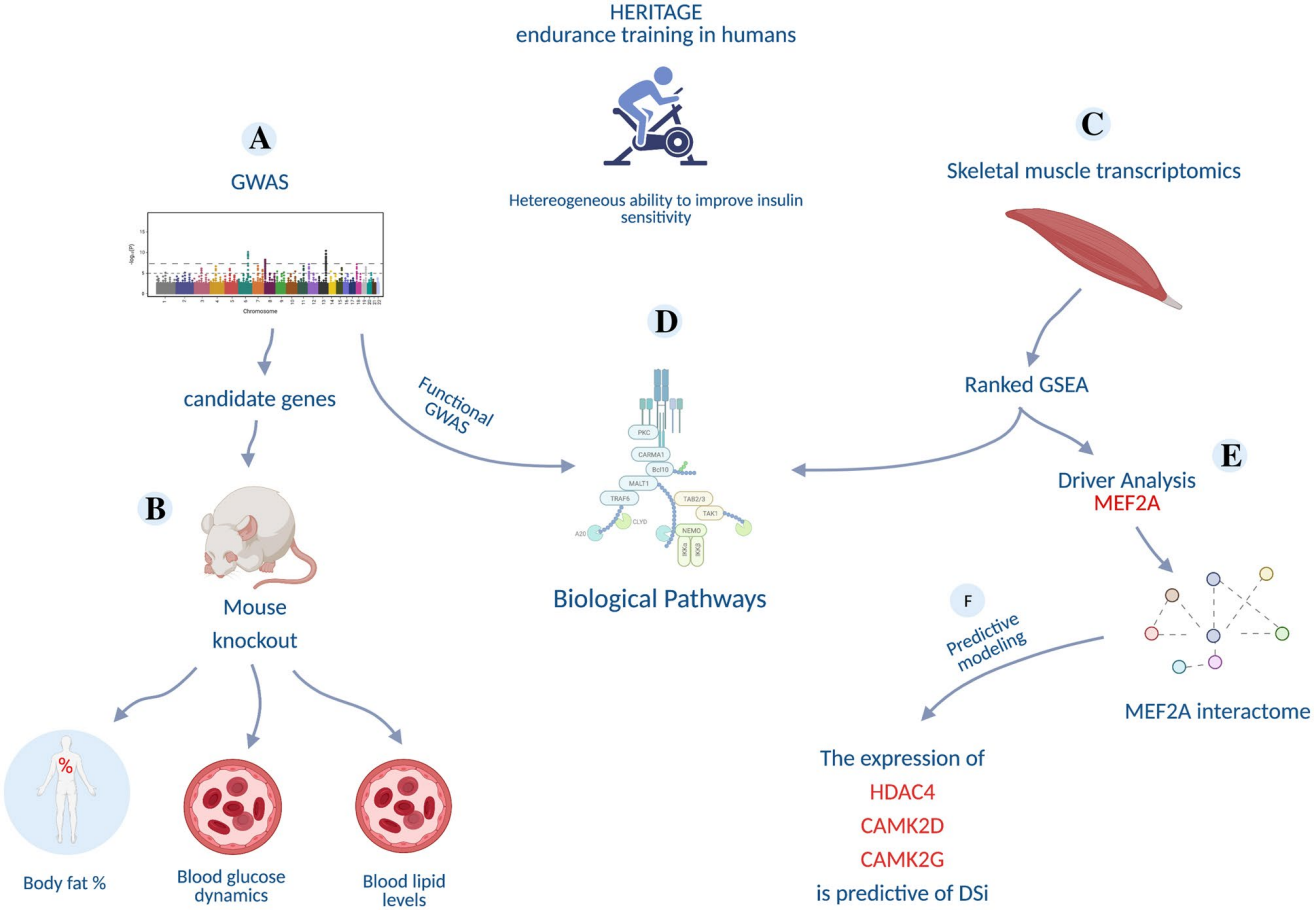
Overview of the study design, consisting of five interconnected steps: (**A**) identification of genetic variants linked to insulin sensitivity index (S_i_), (**B**) validation of the corresponding gene candidates in a mouse knock down experiment database, (**C**) identification of transcriptional signatures in skeletal muscles correlating with baseline S_i_ and ∆S_i_, (**D**) integration of genetics and transcriptomics signatures, (**E**) identification of transcription factors likely to drive the transcriptional signatures linked to ∆S_i_ which led to the identification of MEF2A transcription factor as main driver of the transcriptional profile, and (**F**) development of a statistical model that can predict ∆S_i_ from the transcriptional state of MEF2A interacting genes.

The overarching goal of this study is to investigate the genetic and molecular basis of the variation in S_i_ and ∆S_i_ following EET, across a healthy population. We address this by integrating a traditional GWAS approach with the analysis of skeletal muscle transcriptomics data within HERITAGE, one of the largest studies to evaluate the response of several physiological measurements to EET. The strategy, described in a schematic format in Fig. 1, involved:

1. The identification of genetic variants linked to S_i_ and ∆S_i_ (Fig. 1A) and the validation of the corresponding gene candidates in a mouse knock down experiment database (Fig. 1B).
2. The identification of transcriptional signatures linked to baseline S_i_ and ∆S_i_ (Fig. 1C).
3. The identification of transcription factors that may be driving the transcriptional signatures identified above (Fig. 1E).
4. Testing whether baseline expression of genes linked to TF drivers are good predictors of ∆S_i_ (Fig. 1F).

In addition, by mapping gene candidates identified by GWAS and the transcriptional signatures we tested the hypothesis that genetic variation may be linked to downstream changes in gene expression (Fig. 1D).

### GWAS analysis identifies putative loci linked to baseline and exercise-induced changes in Si

Investigation of genetic variants linked to inter-individual heterogeneity of ∆S_i_ is based on the assumption that its underlying molecular mechanisms have a genetic component. Our analysis revealed that this assumption is indeed likely to be correct as 29% of the variance in ∆S_i_ is accounted for by family membership (Supplementary Fig. S1). Moreover, from an ANOVA, there is 40% more variance between families than within families (*p* = 0.02), providing additional suggestive evidence that the changes in S_i_ in response to exercise training are characterized by a significant heritable component.

Therefore, we set to identify specific genetic variants linked to S_i_ and ∆S_i_ by GWAS. As a first step we tested whether relevant physiological variables may be confounding factors that should be considered in the analysis. We discovered that variation in baseline S_i_ was significantly linked to BMI, VO_2_max/kg, sex and age (Supplementary Fig. S2A). We also discovered that ΔS_i_ was significantly linked to baseline S_i_ and sex (Supplementary Fig. S2B), with 27% of the variance in ΔS_i_ explained by baseline S_i_ (Supplementary Fig. S2C). A similar amount of variance in post-training S_i_ was also explained by baseline S_i_ (Supplementary Fig. S2D). Based on this evidence, we performed a GWAS with the objective to identify SNPs linked to baseline S_i_ and ∆S_i_, where both traits were adjusted for potential confounding variables.

**Figure 2:**
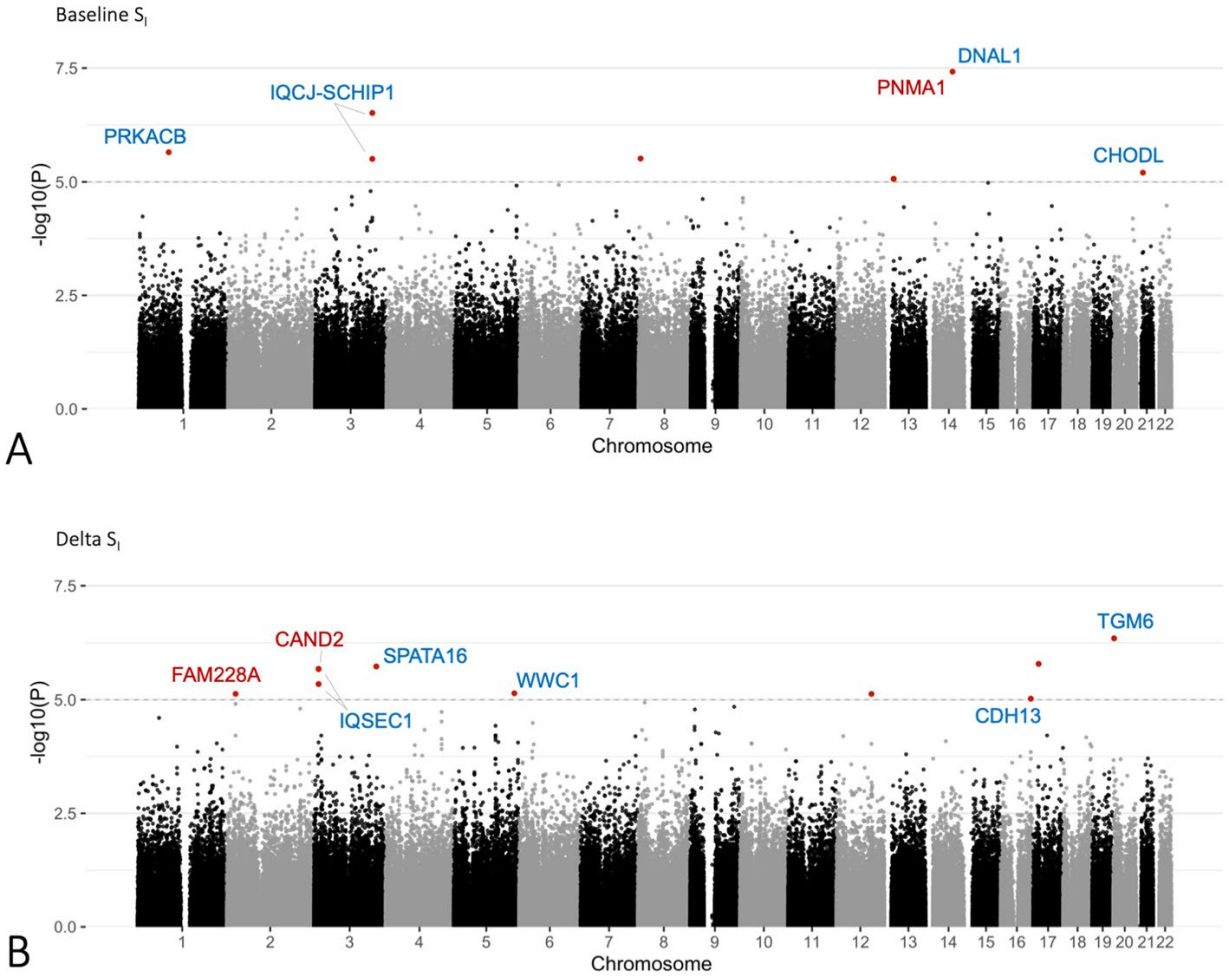
Manhattan plots showing genetic loci associated with baseline and ∆S_i_ and their mapped genes. Genetic loci reaching at least suggestive association are represent by points highlighted in red. Genes mapped to these loci are annotated, where genes mapped by position are highlighted in blue, and genes mapped by eQTL are highlighted in red. Highlighted loci showing no gene annotation did not map to any genes according to the criteria we used. For additional details, see Table 1.

The GWAS analysis identified one SNP significantly associated to baseline S_i_ (*DNAL1* rs11622678, p = 3.79 × 10^−8^) plus seven SNPs with suggestive association (p < 10^−5^), and ten SNPs with suggestive association (p < 10^−5^) with ΔS_i_ (Fig. 2). Positional and eQTL mapping revealed genes that are located within or near these SNPs (± 20 kb window) and/or have their expression correlated with them (Table 1).

**Table 1:** Genomic loci showing, at a minimum, suggestive association with ΔS_i_ and baseline S_i_.

SNP	Chr	Location (bp)	P	Gene symbol	Gene mapping	Gene location (bp)
**Delta S**_**i**_						
rs4815227	20	2441096	4.50E−07	TGM6	Positional	2380908–2432753
rs12449918	17	15392862	1.63E−06	–	–	–
rs6799845	3	172873569	1.86E−06	SPATA16	Positional	172889357–173141268
rs9211^a^	3	12898047	2.10E−06	IQSEC1	Positional	12897043–13282998
	–	–	–	LOC105376955	Positional	12882360–12885099
	–	–	–	RP11-767C1.2	Skeletal muscle eQTL	12832219–12832728
	–	–	–	CAND2	Skeletal muscle eQTL	12796472–12871916
rs14191^a^	3	12898847	2.13E−06	RP11-767C1.2	Skeletal muscle eQTL	12832219–12832728
	–	–	–	CAND2	Skeletal muscle eQTL	12796472–12871916
	–	–	–	IQSEC1	Positional	12897043–13282998
	–	–	–	LOC105376955	Positional	12882360–12885099
rs2600330	3	12926604	4.52E−06	IQSEC1	Positional	12897043–13282998
rs7722673	5	168417231	7.23E−06	WWC1	Positional	168292060–168472303
rs7595372	2	24409253	7.45E−06	FAM228A	Skeletal muscle eQTL	24175069–24200849
rs969863	12	97202907	7.48E−06	–	–	–
rs422455	16	83729855	9.51E−06	CDH13	Positional	82626794–83796610
**Baseline S**_**i**_						
rs11622678*	14	73655058	3.79E−08	PNMA1	Skeletal muscle eQTL	73711783–73714372
	–	–	–	RP3-414A15.10	Skeletal muscle eQTL	73616700–73633941
	–	–	–	DNAL1	Positional	73644875–73703728
	–	–	–	RP3-414A15.2	Skeletal muscle eQTL	73530152–73530610
rs10936174	3	159198318	3.07E−07	IQCJ	Positional	159069252–159266307
	–	–	–	IQCJ-SCHIP1	Positional	159069252–159897366
rs7524898	1	84167162	2.24E−06	PRKACB	Positional	84077975–84238498
rs10107799	8	6102222	3.07E−06	–	–	–
rs7653174	3	159120808	3.13E−06	IQCJ	Positional	159069252–159266307
	–	–	–	IQCJ-SCHIP1	Positional	159069252–159897366
rs205666	21	18120899	6.27E−06	CHODL	Positional	17819595–18267373
rs9511351^b^	13	24575137	8.56E−06	LOC101927375	Positional	24541663–24567321
rs9553347^b^	13	24557233	8.56E−06	LOC105370295	Positional	24538602–24541913
	–	–	–	LOC101927375	Positional	24541663–24567321

*Statistically significant association.

^a,b^r^2^ > 0.8.

**Figure 3:**
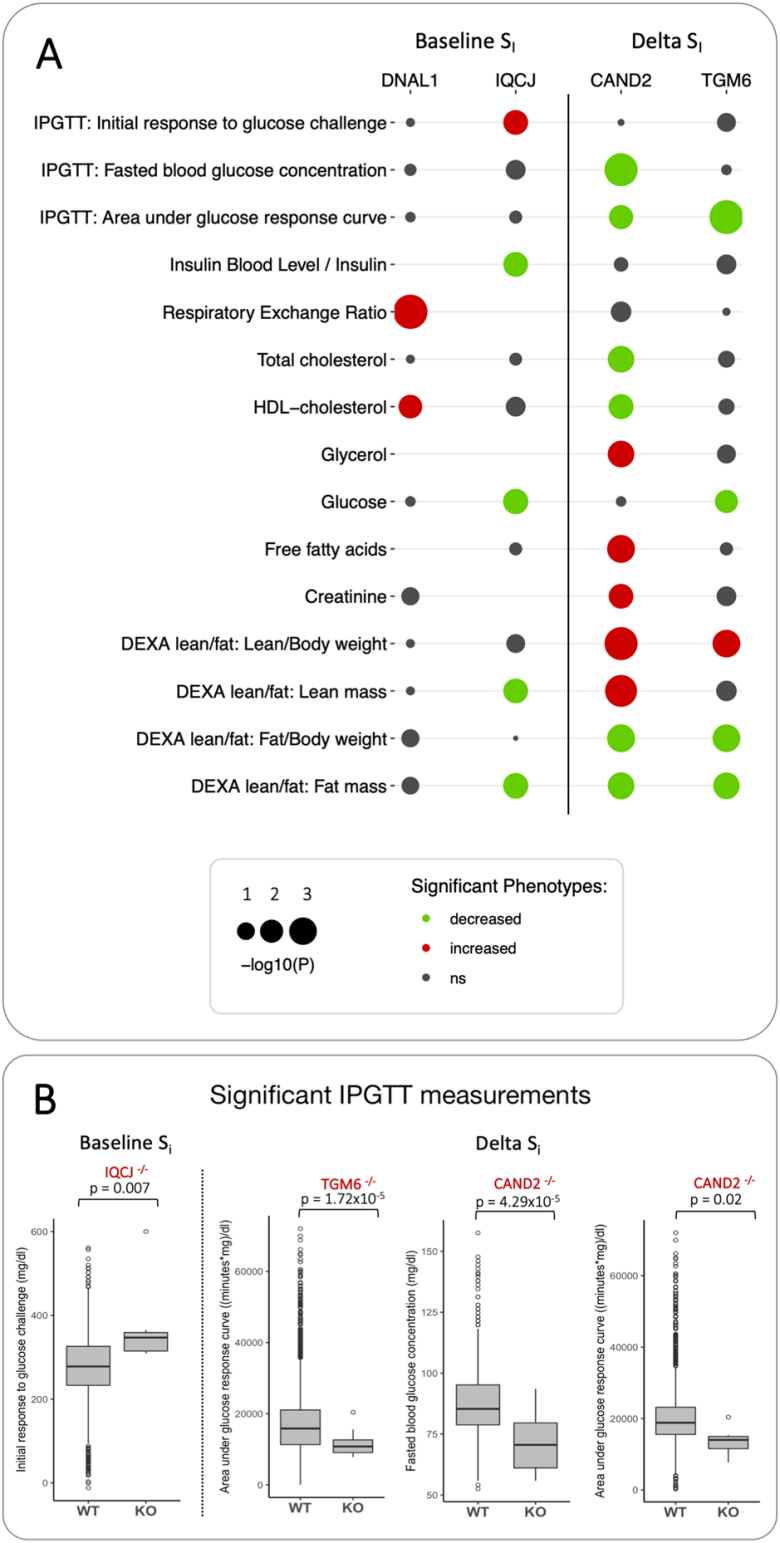
Validation of gene candidates from GWAS analysis in a mouse knock down experiment database. (**A**) Mouse knockout of DNAL1 and IQCJ (associated with baseline S_i_) and CAND2 and TGM6 (associated with ∆S_i_) lead to changes related to metabolism and skeletal muscle development (P_adj_ < 0.05, ns = not significant). Details of each experiment can be found in the IMPC database^18^. (**B**) Boxplots detailing significant associations of IQCJ, CAND2 and TGM6 knockouts with measurements from IPGTT (intra-peritoneal glucose tolerance test) experiment in mice, which is analogous to intravenous glucose tolerance test (IVGTT) method used to measure S_i_ in study participants.

We next tested whether these candidate genes may be causally linked to insulin-regulated physiology. We used a publicly available dataset from the International Mouse Phenotyping Consortium database^18^. This database is the result of an international effort involving 19 assessment centres and reporting a wide range of physiology measurements from a collection of 6440 gene knockouts. Here we selected a panel of physiological measurements of relevance to S_i_ from mouse knockouts in our putative gene lists (see methods section and Fig. 3 for a full list). Eight out of our twelve genes identified by the GWAS (67%) were represented in this database and had a full set of the selected measurements. These genes are PNMA1, DNAL1, IQCJ, CHODL for baseline S_i_ and TGM6, SPATA16, CAND2, CDH13 for ΔS_i_). We found that *DNAL1* and *IQCJ* (linked to baseline S_i_), and *TGM6* and *CAND2* (linked to ΔS_i_) had significant associations with one or more selected mouse phenotypes (Fig. 3).

The *CAND2* knockout is characterized by lower glucose levels, improved glucose tolerance, lower levels of total and HDL cholesterol, increased levels of glycerol, free fatty acids and plasma creatinine, increased lean mass, and decreased fat mass. Interestingly, phenotypes associated with *TGM6* knockout were found to be in accordance with those found associated with *CAND2*, leading also to lower levels of plasma glucose, improved glucose tolerance, lean mass increase, and fat mass decrease. It is worth noting that *CDH13*, *CHODL*, *PNMA1*, and *SPATA16*, although present in the database and tested for relevant phenotypes did not show significant associations with phenotypes relevant to S_i_.

### Functional analysis of the genomic landscape associates the calcium signalling and cholinergic synapse pathways to S_i_

**Figure 4:**
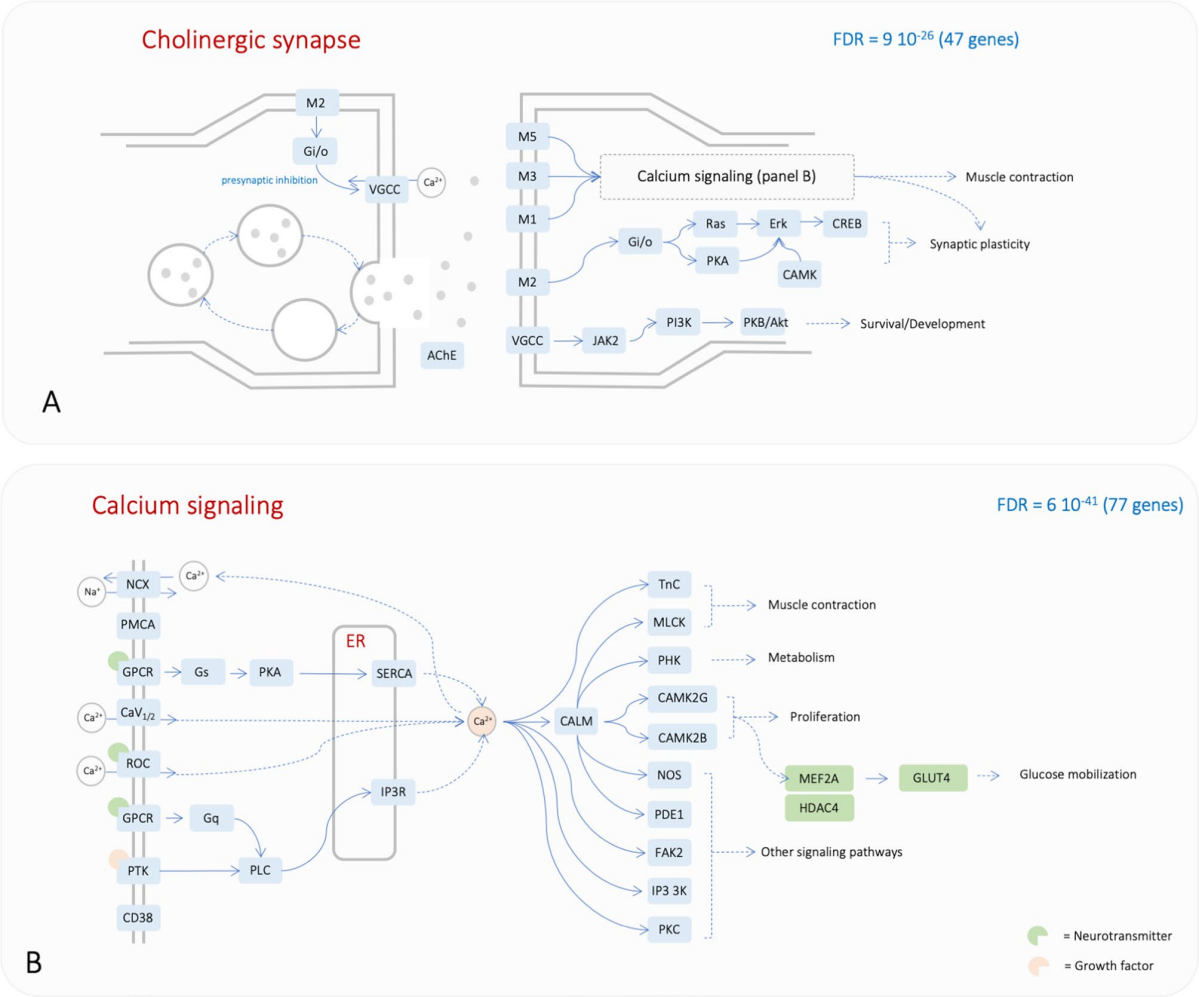
Most significant pathways identified by functional GWAS. (**A**) cholinergic synapse pathway and (**B**) calcium signalling pathway. The two KEGG pathways are interconnected (**B**). The KEGG pathway calcium signalling has been edited to add three additional genes of relevance (green rectangles). Each node can represent multiple genes. Symbols in the two panels are the same as in the original KEGG pathway map (NCX: Na/Ca exchanger; PMCA: ATPase plasma membrane Ca2 + transporting; CaV12: calcium voltage-gated channel subunit alpha1 A and C; ROC: nicotinic acetylcholine receptor alpha-7; GPCR: cysteinyl leukotriene receptor 1; PTK: epidermal growth factor receptor; CD38: ADP-ribosyl cyclase 1; PLC: phosphatidylinositol phospholipase C; Gs: guanine nucleotide-binding protein G(s) subunit alpha; Gq: guanine nucleotide-binding protein subunit alpha-11; SERCA: P-type Ca2 + transporter type 2A; IP3R: inositol 1,4,5-triphosphate receptor type 1; TnC: troponin C; MLCK: myosin-light-chain kinase; PHK: phosphorylase kinase alpha/beta subunit; NOS: nitric-oxide synthase; PDE1: calcium/calmodulin-dependent 3',5'-cyclic nucleotide phosphodiesterase; FAK2: focal adhesion kinase 2; IP3 3 K: 1D-myo-inositol-triphosphate 3-kinase; M1: muscarinic acetylcholine receptor M1; M2: muscarinic acetylcholine receptor M2; M3: muscarinic acetylcholine receptor M3; M5: muscarinic acetylcholine receptor M5; VGCC: calcium voltage-gated channel subunit alpha1 C; AChE: acetylcholinesterase; Gi/o: G protein subunit beta 5; CAMK: CAMK2G and CAMK2B; CREB: cAMP responsive element binding protein 3; PKB/Akt: AKT serine/threonine kinase 3). Genes shown in this figure map to SNPs with *p* value < 0.05.

Complex traits are often characterised by a relatively small contribution of multiple genetic variants that all together contribute to the phenotype of interest. Traditional univariate GWAS analysis, which is often underpowered, may fail to identify these complex interactions. Functional GWAS analysis addresses this issue by testing whether genetic variants tend to cluster within given biological pathways. With this in mind, we analysed our GWAS results with GLOSSI, one such approach. After pruning GWAS results to select independent SNPs (R^2^ < 0.8) and positionally mapping those to genes (± 20 kb window), we identified significantly enriched biological pathways (KEGG pathways and curated skeletal muscle pathways^22^) for both baseline S_i_ and ΔS_i_. This analysis initially identified 17 significant pathways linked to baseline S_i_ and 8 pathways specifically linked to ΔS_i_ (Supplementary Fig. S3). The 17 pathways linked to baseline S_i_ included 470 genes linked to genetic variants with a lower nominal *p* value (*p* < 0.05). Interestingly, we saw that the four most significantly enriched pathways were sufficient to represent half of the 470 most significant genes. These pathways were *calcium signalling* (77 genes), *axon guidance* (63 genes), *chemokine signalling* (66 genes) and *cholinergic synapse* (47 genes) (Fig. 4, Supplementary Fig. S4 and Supplementary Table S2).

The same analysis performed with the top-most significant genes within the pathway enrichment set for ΔS_i_ identified only 31 genes. While this limited number of genes preclude a systematic pathway enrichment analysis, 21 genes could be mapped within the *adrenergic signalling in cardiomyocyte* pathway. Importantly, five of these genes also mapped within the *calcium signalling* pathway (*RYR2*, *SLC8A1*, *CACNA1C*, *CACNA1D* and *CACNA1S*), providing a link with the analysis performed on the baseline S_i_. These included subunits of the ATPase NA+/K+ transporter (ATPA2/A4/B1/B3) and additional calcium voltage-gated channels**.** In addition, there were four subunits of the cytochrome C oxidase enzyme (COX4I2, COX6B1, COX7A1, COX7A2L).

### Transcription factor driver analysis identifies the calcium dependent transcription factor MEF2A as the most significant driver of the ΔSi transcriptional signature

The results of the functional GWAS suggest a role of skeletal muscle in insulin dependent glucose uptake and the effects of exercise in remodelling this tissue. Therefore, we set to investigate the transcriptomic profile of skeletal muscles in a subset of the HERITAGE individuals. We wanted to identify baseline transcriptional signatures that correlate to S_i_ and ΔS_i_ as well as the transcription factors that may drive such signatures. More specifically, by using a GSEA based approach we searched for enrichment in transcription factor binding sites in the list of genes correlated to S_i_ and ΔS_i_.

Only when including fibre type composition in the models we have identified gene sets (a total of 45) mapped to known transcription factor binding sites significantly enriched by genes whose skeletal muscle expression correlated to ΔS_i_ (Supplementary Table S3). Fibre type composition has been previously linked to insulin-dependent glucose uptake in skeletal muscle^32–34^ and its addition to the ΔS_i_ model also led to a larger number of significantly enriched biological pathways. These encompassed a variety of biological functions such as signalling, energy and amino acid metabolism, tissue homeostasis, protein degradation, immune system, and translation (Supplementary Fig. S5). Interestingly, the KEGG pathways chemokine signalling, neuroactive ligand receptor interaction, and the functional term calcium dynamics/homeostasis required for excitation–contraction coupling were reminiscent of the results for the functional GWAS analysis (Supplementary Table S2).

**Figure 5:**
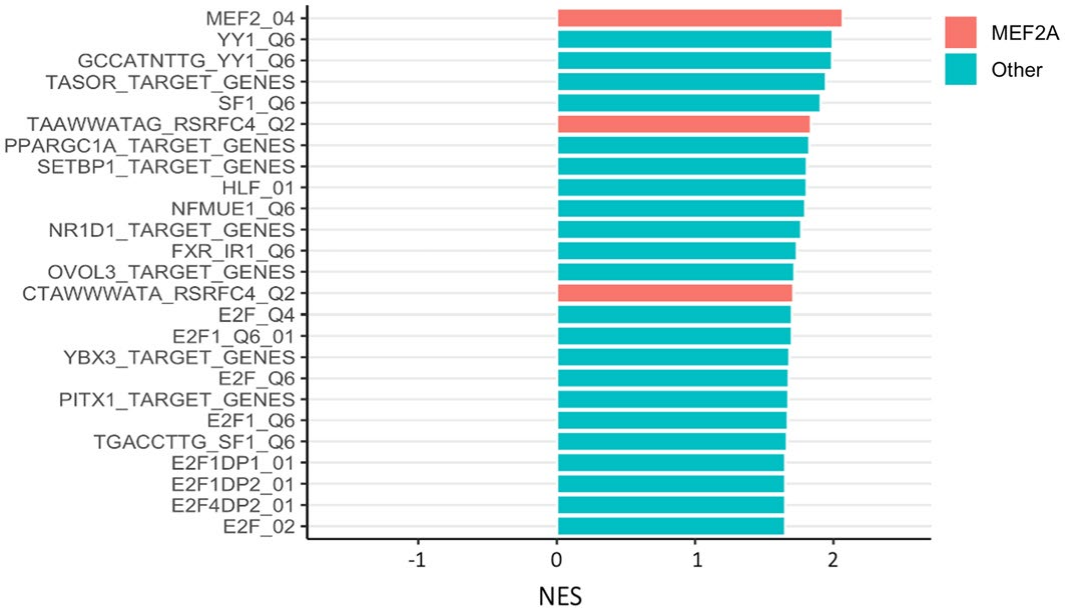
Top 25 transcription factor target gene sets significantly enriched in GSEA (P_adj_ < 0.05) ranked by NES (normalised enrichment score). Gene sets with a positive NES are enriched by genes with skeletal muscle baseline expression positively correlated to ΔS_i_ while genes with a negative NES are enriched by genes with skeletal muscle baseline expression negatively correlated to ΔS_i_. Sets corresponding to MEF2A target genes are highlighted in red. A full list of significant transcription factor target gene sets is available on Supplementary Table S3.

Remarkably, when we examined which transcription factor may be able to explain the expression of genes in the S_i_ transcriptional signatures, we found that the top candidate gene was the calcium dependent transcription factor MEF2A (Fig. 5). The hypothesis that MEF2A drives a significant part of the transcriptional signature linked to ΔS_i_ is supported by the observation that the global transcriptional signature associated to MEF2A knockdown in C2C12 can recapitulate the transcriptional signature correlated to ΔS_i_ in the HERITAGE cohort (Fig. 1, Supplementary Information and Supplementary Table S4). This result is consistent with the findings from the functional GWAS that suggested a key role of calcium signalling in baseline S_i_. The linkage between genetic variation and the transcriptomics signatures in skeletal muscle associated with S_i_ emerged through the linkage between calcium signalling and MEF2A.

### The development and validation of a baseline MEF2 transcriptional signature predictive of ΔS_i_

Ranked GSEA analysis revealed that genes with baseline expression levels showing higher (positive) or lower (inverse) correlation with ΔS_i_ were found to be enriched by MEF2A targets (Supplementary Fig. S6 and Supplementary Table S5). We also show that the transcription factor MEF2A may be the main actor driving that transcriptional signature. We therefore set to develop a predictor of ΔS_i_ based on the baseline expression of genes that are part of the MEF2A interactome. We focused on 50 experimentally validated interactors with a high confidence score (identified in the STRING database, see Supplementary Table S1 for an exhaustive list) and we performed a comprehensive analysis of all possible linear regression models based on gene expression levels of all possible combinations of three-genes sets (a total of 19,600 models). Remarkably, the most predictive statistical model (R^2^ = 48%, F-value = 6.4, *p* < 0.001; Table 2) included the direct activators of the MEF2 transcription factor (*HDAC4*, *CAMK2D,* and *CAMK2G,* Fig. 4B). In comparison, a linear regression model comprising only of sex and VO2max/kg as predictors showed poor predictive power (R^2^ = − 0.01%, F-value = 0.98 and *p* = 0.39), reinforcing the large contribution of these genes on predicting ΔS_i_. Consistent with the established hetero-multimeric nature of CaMKII, the model did show a highly significant interaction between the two isoforms *CAMK2D* and *CAMK2G*. Regression diagnostics confirmed conformity of the residuals to the assumptions of normality, linearity and homoscedasticity.

**Table 2:** Result of the mRNA-based multivariate regression model for S_i_ training response among the subset of White HERITAGE participants for which global gene expression data before and after the exercise program are available (n = 47).

Variable	Regression coefficient	Standard error	*t*-value	*p* value
VO_2max_	− 0.03	0.004	− 0.68	0.50
Sex	− 2.15	0.83	− 2.59	0.013
CAMK2D mRNA	40.73	27.98	1.46	0.15
CAMK2G mRNA	74.78	32.04	2.33	0.02
HDAC4 mRNA	− 34.94	22.37	− 1.56	0.13
CAMK2D:CAMK2G	− 12.08	3.41	− 3.54	0.001
CAMK2D:HDAC4	6.94	1.63	4.27	< 0.001
CAMK2G:HDAC4	− 0.40	2.90	− 0.14	0.89

Importantly, the response variable (i.e. ∆S_i_) spanned a broad range (− 79 to + 120%) among the participants included in the current analysis. Sex and VO_2_max adjusted for body size were included as covariates in the model. Notably, the abundance of these transcripts was not responsive to the training intervention, but rather higher basal expression levels were associated with greater gains in S_i_. CAMK2D: 228555_at; CAMK2G: 212757_s_at; HDAC4: 228813_at.

To test the general applicability of the HERITAGE predictive gene signature, we took advantage of a previously published Affymetrix muscle gene expression dataset from a smaller independent mixed exercise training cohort^35^. Importantly, this cohort also spanned a broad range in terms of the training-induced change in the rate of peripheral glucose disappearance (Rd) (∆Rd ranging from − 20 to + 145%). Intriguingly, the baseline multi-gene RNA signature was able to explain 30% of the training-induced change in Rd among the healthy middle-aged male participants (n = 14) in the replication cohort (see Supplementary Fig. S7, a value close to the estimated ∆S_i_ variance in the HERITAGE cohort when family membership has been accounted for).

## Discussion

Here, we have shown that variation in insulin sensitivity across a normal healthy population and its modulation by EET is a complex trait where combined variation in genes linked to the KEGG pathways *cholinergic signalling*, *calcium signalling, axon guidance and chemokine signalling* is likely to be an important component. Despite such complexity, we have been able to identify genes that are causally linked to glucose disposal and other relevant phenotypic traits in mouse knockouts.

### GWAS candidate genes associated to baseline and ∆S_i_

Our study is the first to investigate genome-wide associations with changes in S_i_ in response to exercise training. Despite the SNPs we identified in the present study as significantly associated to baseline S_i_ have not been previously associated to insulin sensitivity according to GWAS Catalogue database^36^, some of them have been previously linked to insulin resistance or related phenotypes. Importantly, mouse knockouts for four of these candidates (*TGM6*, *CAND2*, *IQSEC1* and *DNAL1*) showed a relevant phenotype, suggesting a causal link with S_i_. Here we review the evidence in the literature that is consistent with our findings.

The function of *TGM6* product, transglutaminase 6 (TG6), has not been studied extensively and is not yet well understood. However, transglutaminase 2 (TG2) has been implicated in glucose metabolism^37,38^ and glucose tolerance^39^. Transglutaminases catalyse serotonylation^40^, a process involved in the modulation of insulin secretion in pancreatic beta-cells^41^. The results of the *TGM6* knockout experiment and the function of TG2 suggest a role for TG6 in S_i_ and warrants further investigation.

There are several pieces of evidence that are consistent with a role of *CAND2* in insulin sensitivity. CAND2 is mostly expressed in muscle tissues^17,42^. Beyond its role in myogenic differentiation^43–45^, CAND2 interacts with insulin receptor substrate 1 (IRS1) and is stimulated by insulin in type 2 diabetes patients, but not in non-diabetic controls^46^. CAND2 acts by modulating the assembly of ubiquitin–proteasome related complexes, such as E3 ligases^47,48^, which also have been implicated in insulin resistance and diabetes and are known to target key insulin signalling molecules^49^. Moreover, *CAND2* has been shown to be upregulated during a 3 h hyperinsulinemic euglycemic clamp in vastus lateralis muscle of healthy subjects^50^. *CAND2* has been previously mapped to SNPs associated with related phenotypes such as waist-hip ratio^51^ and waist circumference adjusted for BMI^52,53^.

SNP rs11622678 located in chromosome 14 reached a statistically significant association with baseline S_i_ and was positionally mapped to *DNAL1.* Mutations in this gene cause primary ciliary dyskinesia as this gene affects movement of cilia and flagella^54^, and have been associated with respiratory diseases and lung function^18,51^. Interestingly, knockout of this gene led to increased respiratory exchange ratio (RER), with higher values indicating that carbohydrates are the main source of substrates being oxidized (Fig. 3).

Two SNPs (r^2^ = 0.49) have been positionally mapped to the fusion transcript *IQCJ-SCHIP1* spanning two adjacent independent genes. Although the functions of *IQCJ-SCHIP1* are still poorly understood, genetic variants tagging *IQCJ* have been associated to modulation of blood lipid levels in multiple independent studies^55–58^, while *SCHIP1* has been implicated in axon guidance^59–61^ and was upregulated in differentiated myotubes compared to undifferentiated^62^ (see Supplementary Material for additional discussion on PNMA1, CHODL, SPATA16 and CDH13 which are either not present in the IMPC database or no relevant traits showed significant changes following knockout).

Furthermore, the observation that baseline S_i_ negatively correlates with ∆S_i_ (Supplementary Fig. S2C) is intriguing and suggestive of the existence of an upper limit for S_i._ This would result in a lower margin for improvement in individuals with a high S_i_ value.

### Is calcium mobilization triggered by muscle contraction potentially responsible for changes in S_i_?

In addition to the genes discussed above, we identified a consistent accumulation of SNPs correlated to baseline S_i_ and ΔS_i_ in *calcium signalling* and *cholinergic synapse* pathways (Supplementary Table S2). These findings suggest that cholinergic signalling via mobilization of calcium in skeletal muscle may mechanistically link muscle contraction to insulin sensitivity. A study investigating correlation between baseline gene expression and exercise-induced %ΔS_i_ found that several significantly correlated genes in a validation cohort mapped to Ca^2+^ signalling, including *CACNA1S* and *CAMK2D*^63^.

There is considerable evidence that insulin signalling and muscle contraction are linked processes that activate multiple signalling cascades leading to glucose uptake^64–67^. Glucose uptake by skeletal muscle tissue is mediated by GLUT4, which upon stimulation by either insulin and/or contraction is translocated to the plasma membrane from vesicles. In insulin-mediated glucose uptake, insulin binding triggers a cascade of molecular reactions that lead to GLUT4 translocation, also triggering a transient Ca^2+^ influx in muscle cells. This process is suggested to enhance GLUT4 translocation and docking in the plasma membrane. Meanwhile, muscle contraction initiated by membrane depolarisation and increased concentrations in intracellular Ca^2+^ leads to activation of Ca^2+^ sensors such as Ca^2+^-calmodulin-dependent kinase II (CAMKII), which are key molecules in contraction-stimulated glucose transport. Activated CAMKIIs promote dissociation of HDAC4 from MEF2A transcription factor, leading to its activation and increased GLUT4 transcription^68,69^. Therefore, insulin- and contraction-mediated signalling pathways related to skeletal muscle glucose uptake are inter-twined, where GLUT4 increased expression and availability entrained by muscle contraction would also contribute to improved insulin-mediated glucose uptake.

It is therefore conceivable that variation in genes controlling muscle contraction (represented in the cholinergic synapse and calcium signalling pathways) could result in lower levels of intracellular Ca^2+^, leading to lower activation of the CAMKIIs and consequently reduced GLUT4 availability (Fig. 4B). Diminished localization of GLUT4 to the membrane, which is also Ca^2+^ dependent, could also contribute to glucose uptake impairment in skeletal muscle. Interestingly, none of the analyses shown here directly linked *SCL2A4* (gene encoding for GLUT4) to either baseline or ΔS_i_. It is possible that our study, is not sufficiently well powered to capture *SCL2A4* effect size, or that other mechanisms affecting GLUT4 regulation, such as post-translation modifications^70^, modulate insulin sensitivity. Additionally, none of the publicly available GWAS studies on insulin sensitivity (GWAS Catalog database^36^) reported associations with SLC2A4.

Genetic variation may not be the only mechanism controlling glucose mobilization and ultimately S_i_. A study investigating epigenetic patterns associated to type 2 diabetes has shown that first-degree relatives of patients with diabetes have differential DNA methylation patterns in genes related to insulin and Ca^2+^ signalling pathways compared to healthy individuals with no family history of the disease. Intriguingly, DNA methylation of genes involved in Ca^2+^ signalling pathways including *MEF2A*, which we also have identified in our approach, decreased after exercise^71^. The role of genetic and epigenetic variation in Ca^2+^ signalling in modulating inter-individual variability in insulin sensitivity warrants further investigation.

### Other potential mechanisms linking genetic variation to S_i_

Our analyses suggest that genetic variation affecting other biological mechanisms could also be modulating S_i_. The chemokine signalling pathway has been identified in both GWAS and transcriptomics functional analyses, suggesting that variation in genes within this pathway could be affecting their expression and contributing to variation in exercised-induced S_i_ response.

Several studies have suggested a role of chemokines and chemokine receptors in the development of insulin resistance, which is attributed in part to a state of low-grade inflammation due to elevated blood glucose and lipid levels induced by diet and excess adiposity^72,73^. This leads to induction of pro-inflammatory mediators such as chemokines that interfere with insulin signalling pathways. In the present study, we have identified gene expression profiles associated to inflammation-related pathways correlated with S_i_ response, suggesting genetic variation affecting chemokine signalling could affect the inflammatory processes that naturally occur with exercise. Additionally, the emerging research field of ‘immunometabolism’^74^ has generated data indicating that a cross‐talk between immune- and metabolic-related molecules is essential to normal skeletal muscle physiology^75^.

Cell adhesion molecules pathway, which include key molecules involved in modulating ECM integrity, was also identified in both GWAS and transcriptomics functional analyses. There is some evidence linking insulin resistance to ECM remodelling^75^ with mechanisms attributed to physical impairment of insulin access to its receptor by increased ECM deposition, or to the roles of integrins in mediating insulin signalling^76^. Deletion of the muscle-specific integrin β1 (expressed by *ITGB1*) results in decreased insulin sensitivity, whereas the ECM of insulin resistant human muscle were reported to be associated to decreased abundance α-actinin 2 (expressed by *ACTN2*). Interestingly, in our functional investigation encompassing ∆S_i_ associated genetic variants, two SNPs mapping to *ACTN2* and *ITGB1* genes are second and third top significant SNPs (*p* < 0.001), but several other top associated SNPs map to cell adhesion molecules, suggesting that mutations in ECM-related molecules could be influencing the S_i_-related traits at rest or in response to regular exercise.

## Conclusions

The relatively large proportion of individuals who fail to improve metabolic fitness traits justify the importance of developing evidence-based personalized exercise prescription to maximize the health-promoting benefits of a physically active lifestyle. To develop such individualized recommendations for exercise, it is vital that the molecular basis driving phenotypic response variation be understood. Our multi-omics approach is a step in this direction as it provides evidence of a genetic component affecting calcium signalling that might be responsible for the large heterogeneity in ∆S_i_ following a fully supervised EET program. The predictive RNA signature can potentially be used to stratify individuals before any intervention has taken place. Further studies are needed to test whether our signature could be predictive of response in different training protocols or whether ∆S_i_ non-responders could benefit from different training regimes (*e.g.* high-intensity interval training or resistance exercise). This is important as skeletal muscle insulin resistance is one of the earliest hallmarks of the development of type 2 diabetes and other metabolic complications. Promisingly, muscle RNA abundance can now be more easily quantified due to the development of less invasive micro-needle biopsy sampling. Further, one-step multiplex real-time RT-PCR assays could offer a rapid, sensitive and cheap diagnostic option if a molecular predictor could be validated and replicated in multiple cohorts.

## Supplementary Information

Supplementary Figures.

Supplementary Information 1.

Supplementary Tables.

## Data Availability

The datasets generated during and/or analysed during the current study are available from the corresponding author on reasonable request.
