# Supplementary material for: Genomics and transcriptomics landscapes associated to changes in insulin sensitivity in response to endurance exercise training
Source: Sci Rep. 2021 Dec 2;11:23314. doi: 10.1038/s41598-021-98792-1 (PMC8639975; doi:10.1038/s41598-021-98792-1)
Supplement: Supplementary file 1 — Supplementary Figures. [file 41598_2021_98792_MOESM1_ESM.pdf]

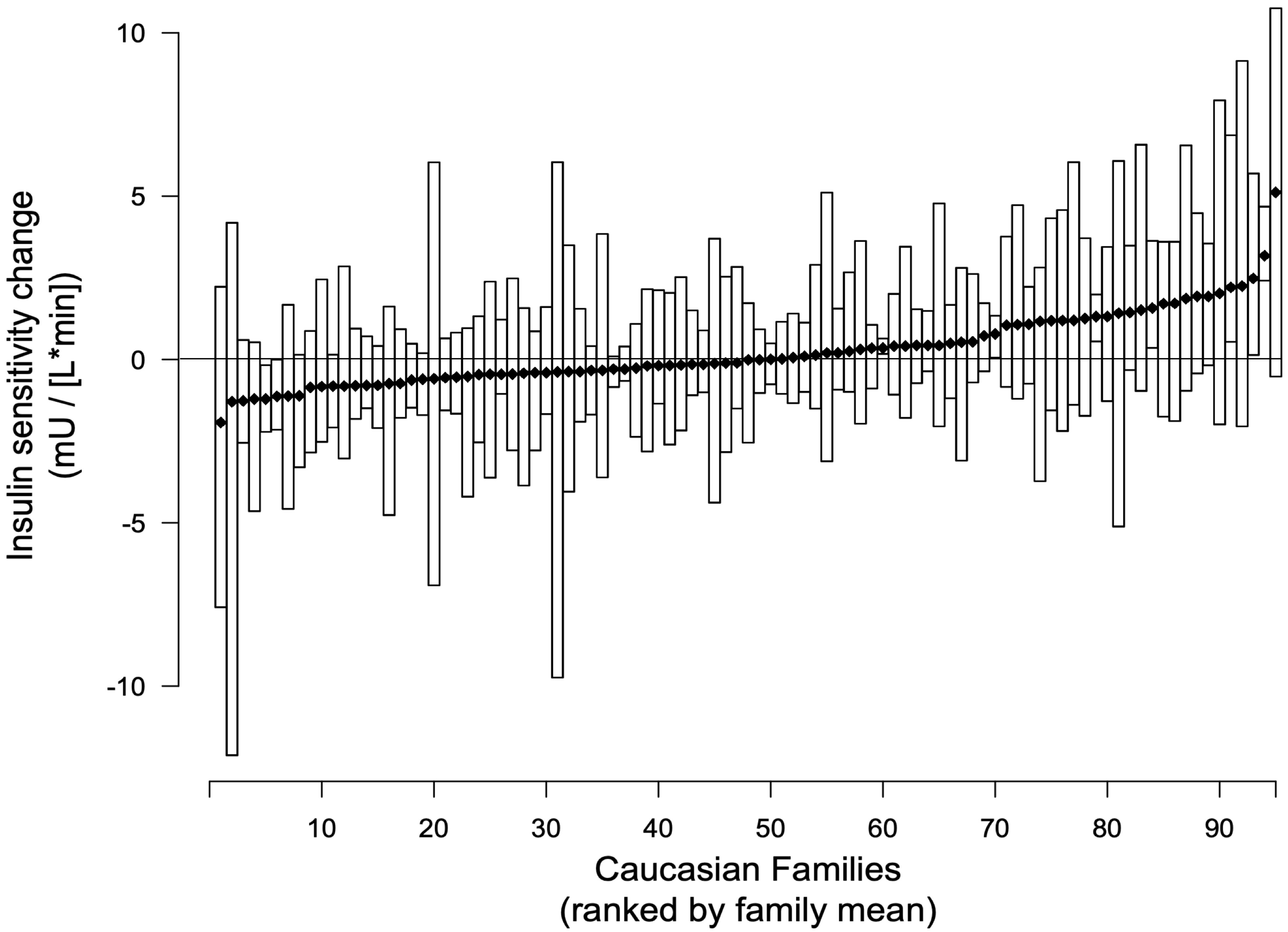

Figure S1: Training-induced change in peripheral insulin sensitivity plotted against Caucasian family rank (*i.e.* families ranked by family mean) in the HERITAGE study. Only families with at least 3 members are shown (94 families in total); family average was 4.3 members (parents and offspring considered). Each vertical box represents the range of training response across all family members (parents and offspring). Darker horizontal reference line denotes family mean. The *F*-value from the ANOVA indicates that there is 40% more variance between than within families (p=0.02), with 29% of the variance being accounted for by family membership.

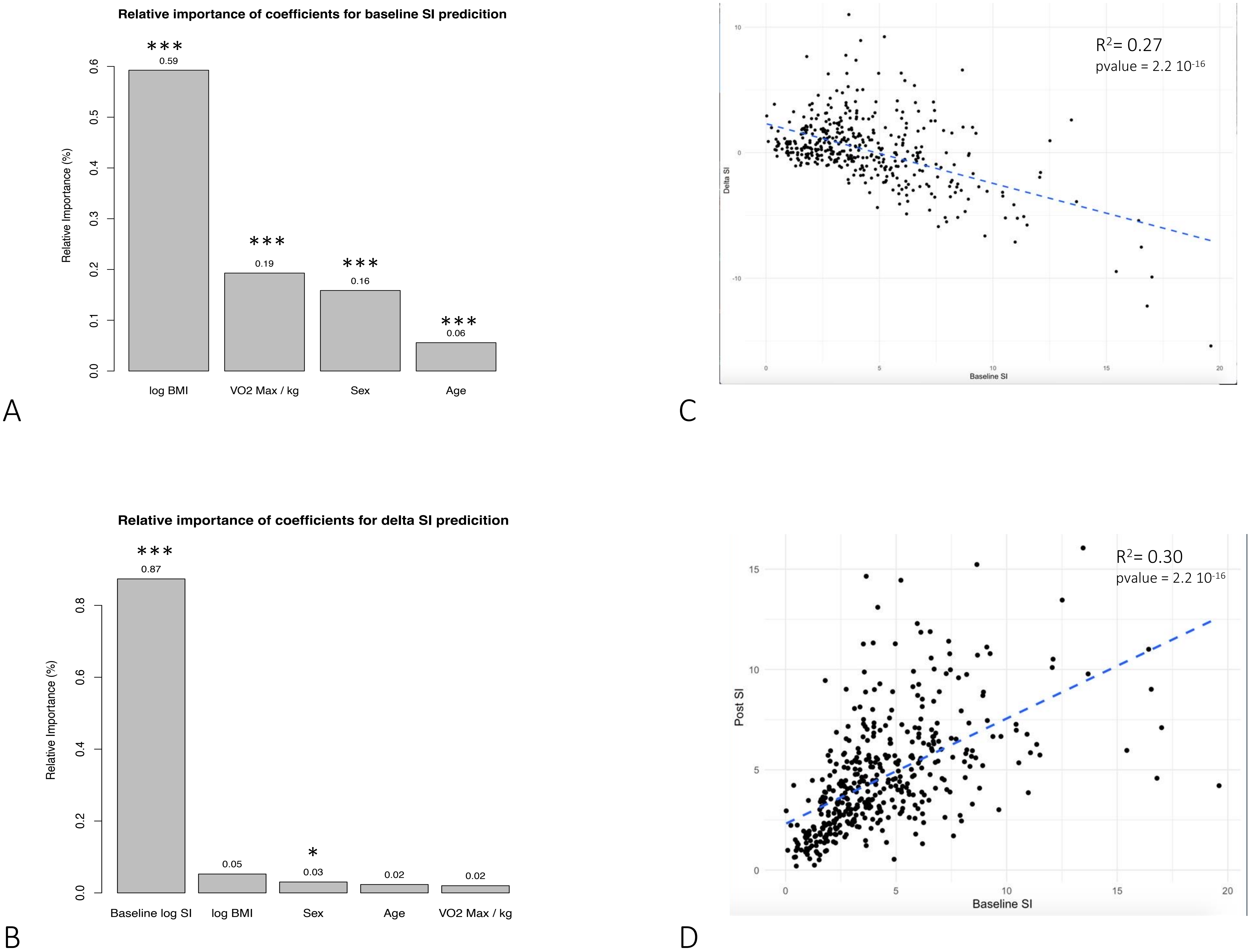

Figure S2: Relative importance of variables in linear models regressing baseline Si (A) and ΔSi (B) are indicated in the the top of each bar and have been calculated using the *lmg* method from relaimpo R package. Statistical significance of each variable was also tested by generating ANOVA models (***P < 0.001, **P < 0.01, *P < 0.05). (C) Linear model regressing ΔSi against baseline Si, (D) Linear model regressing post-training Si against baseline Si.

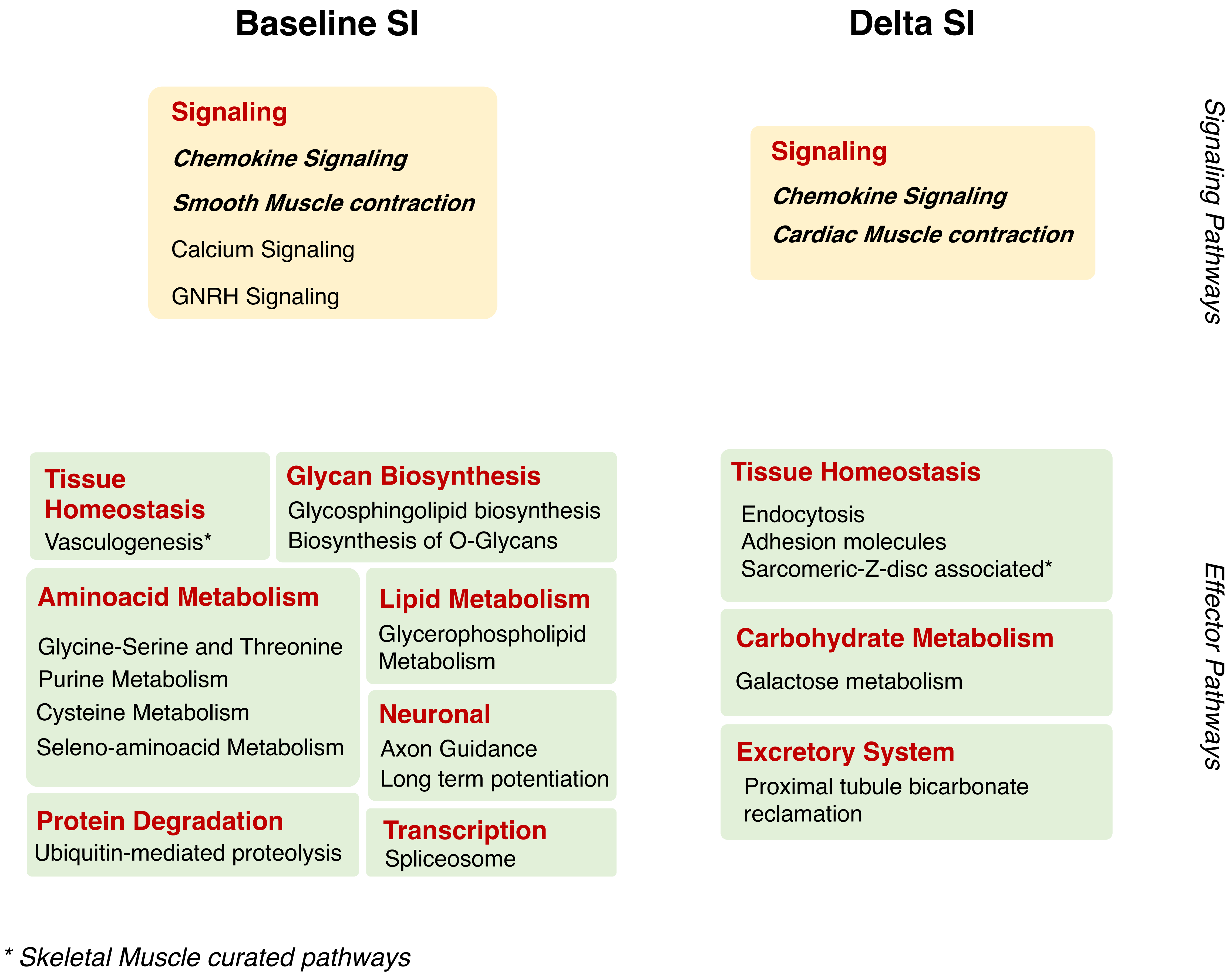

Figure S3: Functional GWAS. This is the result of the GLOSSI analysis including all genes rather than the highly significant ones.

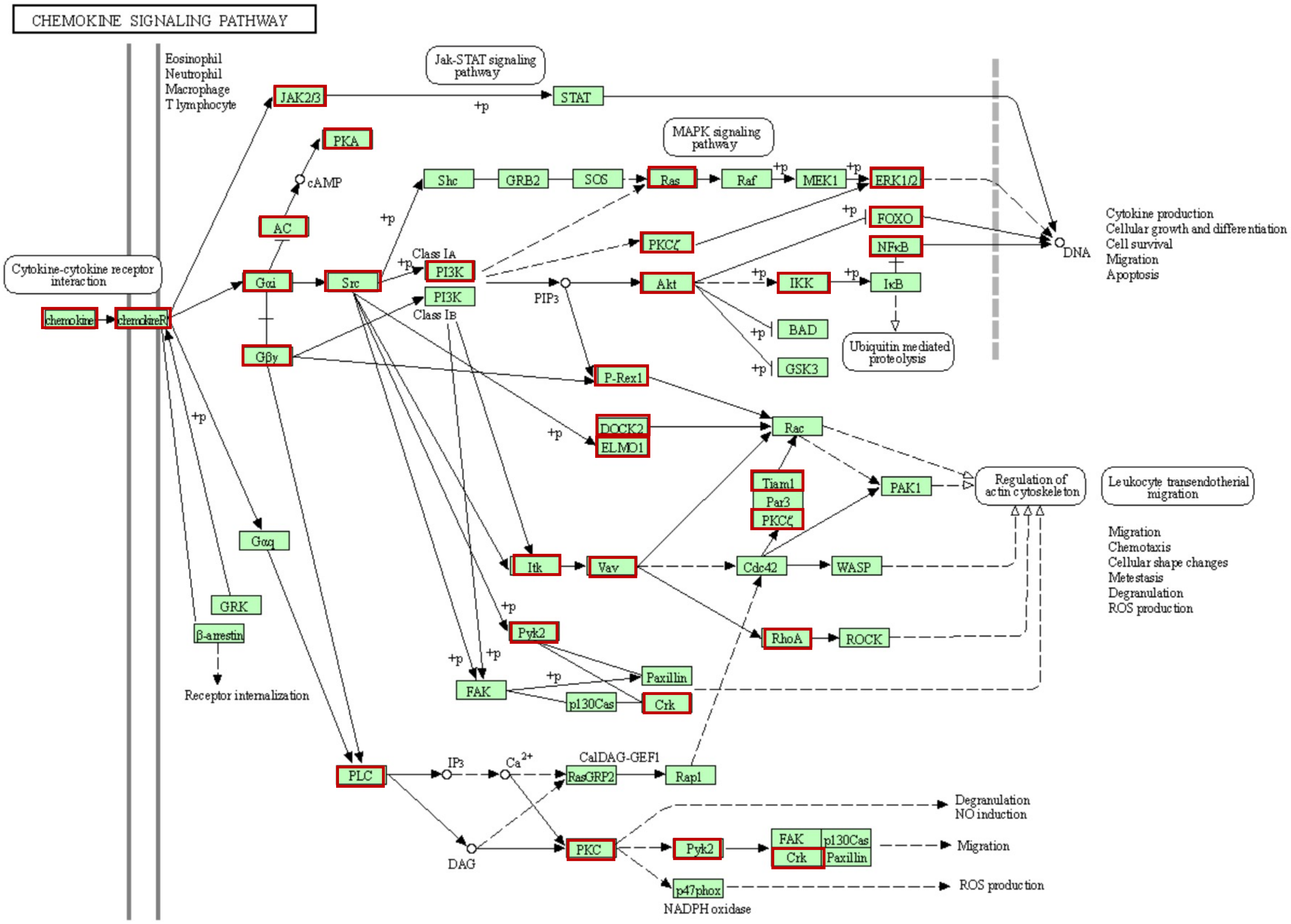
CHEMOKINE SIGNALING PATHWAY
Eosinophil
Neutrophil
Macrophage
T lymphocyte
Jak-STAT signaling pathway
JAK2/3
STAT
+p
PKA
cAMP
AC
MAPK signaling pathway
Shc
GRB2
SOS
Ras
Raf
MEK1
ERK1/2
FOXO
NFκB
Cytokine-cytokine receptor interaction
chemokine
chemokineR
Gαi
Src
Class IA
PI3K
PI3K
Class IB
PKCζ
Akt
PIP3
IKK
IκB
BAD
GSK3
Ubiquitin mediated proteolysis
DNA
Cytokine production
Cellular growth and differentiation
Cell survival
Migration
Apoptosis
Gβγ
P-Rex1
DOCK2
ELMO1
Rac
Tiam1
Par3
PKCζ
PAK1
Regulation of actin cytoskeleton
Leukocyte transendothelial migration
Migration
Chemotaxis
Cellular shape changes
Metestasis
Degranulation
ROS production
Gαq
Itk
Vav
Cdc42
WASP
GRK
β-arrestin
Receptor internalization
Pyk2
RhoA
ROCK
Paxillin
FAK
p130Cas
Crk
PLC
IP3
Ca2+
CalDAG-GEF1
RasGRP2
Rap1
Degranulation
NO induction
DAG
PKC
Pyk2
FAK
p130Cas
Crk
Paxillin
Migration
p47phox
NADPH oxidase
ROS production

A

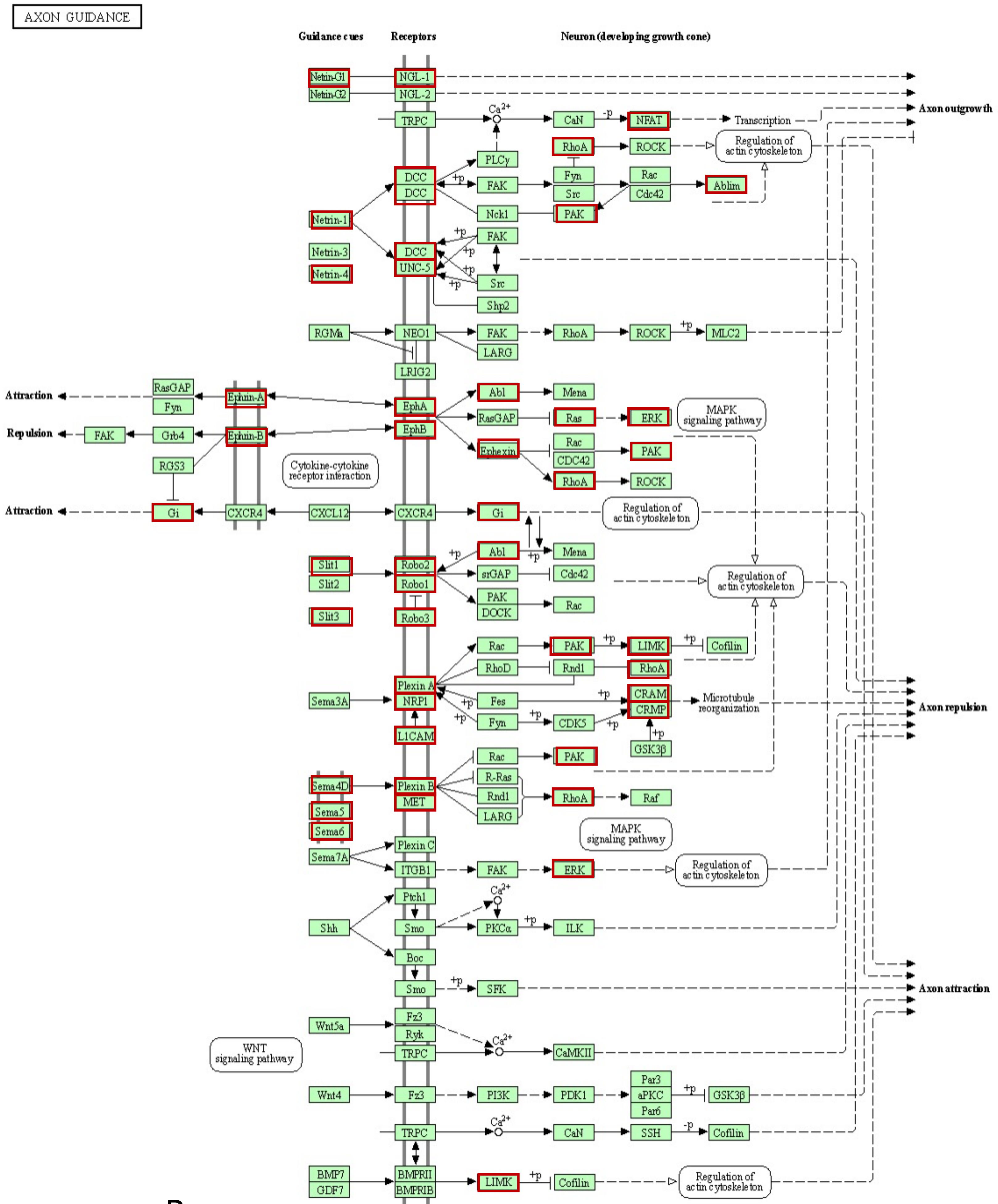

B

Figure S4. Functional pathways enriched in the functional GWAS for baseline Si with highly significant SNPs
The figure shows two of the four most significant pathways enriched in the functional GWAS. Panel A represent the KEGG pathway *Cytokine signaling. P*anel B represents the KEGG pathway *Axon guidance*. Genes are mapped on the pathway with red rectangles. The Figures are obtained by the KEGG database (copyright Kanehisa laboratory).

Delta SI (adjusted for Fiber Type %)

Signaling Pathways

Signaling

Olfactory Transduction
Toll-like Receptor Signaling Pathway
Fc Epsilon Ri Signaling Pathway
B Cell Receptor Signaling Pathway
Chemokine Signaling Pathway
Cytokine-Cytokine Receptor Interaction
Neuroactive Ligand Receptor Interaction
Jak Stat Signaling Pathway
Taste Transduction
Calcium dynamics / homeostasis required for ECC*

Effector Pathways

Energy Metabolism

Citrate Cycle TCA Cycle
Oxidative Phosphorylation
Butanoate Metabolism
Propanoate Metabolism
Pyruvate Metabolism
Glyoxylate And Dicarboxylate Metabolism
Oxidative Metabolism*
Glycolytic Metabolism*
Mitochondrial Electron Transporters*

Tissue Homeostasis

Focal Adhesion
Cell Adhesion Molecules
Regulation of Actin Cytoskeleton
Fc Gamma R Mediated Phagocytosis
Extracellular Matrix*

Translation

Aminoacyl tRNA Biosynthesis

Protein Degradation

Ubiquitin-mediated proteolysis

Aminoacid Metabolism

Valine Leucine And Isoleucine Degradation
Arginine And Proline Metabolism
Valine Leucine And Isoleucine Biosynthesis
Seleno-amino Acid Metabolism

Immune System

Hematopoietic Cell Lineage
Leukocyte Transendothelial Migration
NK Cell Mediated Cytotoxicity
Complement And Coagulation Cascades
Antigen Processing And Presentation
Inflammation*

Figure S5: Transcriptional analysis of DSI associated transcriptome by GSEA

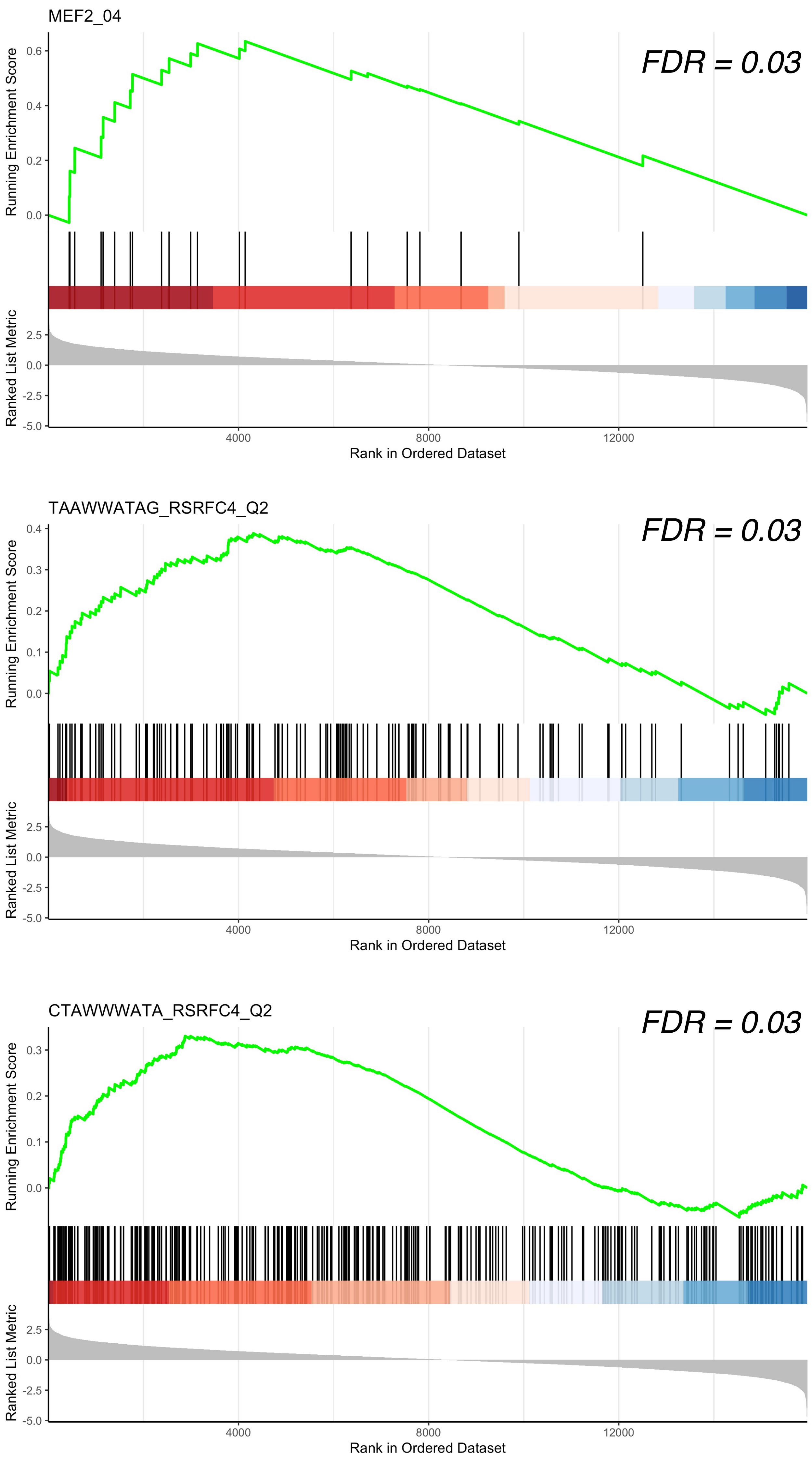

Figure S6: Enrichment score (ES) plots of MEF2A target gene sets showing over-representation of MEF2A targets on the positive end of the ranked gene list (FDR < 0.05). Ranking is based on T-values from linear mixed models regressing delta Si with baseline mRNA levels and predictors shown as Ranked List Metric. Positive ES values point to enrichment among genes positively correlated with delta Si. Vertical bars refer to individual genes in a gene set and their position reflects the contribution of each gene to the ES. Genes that belong to the leading edge (i.e. genes that appear before or at the maximum ES contribute to the enrichment signal (Supplementary Table S4).

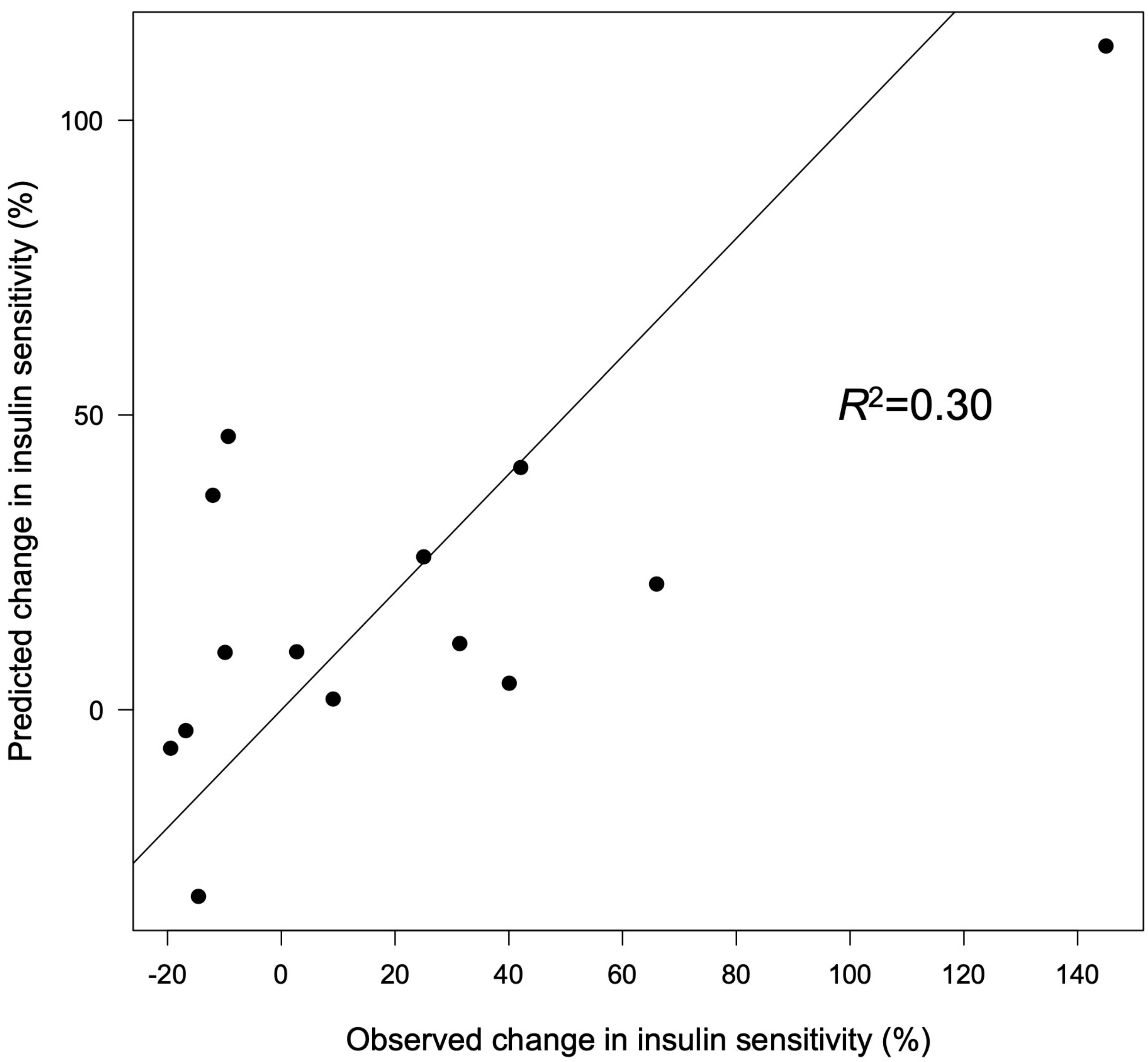

Figure S7: Performance of the mRNA-based regression model derived from the HERITAGE study in an independent training cohort. In this study, peripheral glucose disappearance (Rd) was measured using the hyperinsulinemic-euglycemic clamp technique. Scatter plot of experimentally determined (observed) changes in peripheral insulin sensitivity versus predicted values for training-induced changes in peripheral insulin sensitivity (n=14, r^2=0.30).
