# Supplementary material for: Genomics and transcriptomics landscapes associated to changes in insulin sensitivity in response to endurance exercise training
Source: Sci Rep. 2021 Dec 2;11:23314. doi: 10.1038/s41598-021-98792-1 (PMC8639975; doi:10.1038/s41598-021-98792-1)
Supplement: Supplementary file 2 — Supplementary Information 1. [file 41598_2021_98792_MOESM2_ESM.docx]

**Supplementary Information**

**Genomics and transcriptomics landscapes associated to changes in insulin sensitivity in response to exercise**

Louise Y. Takeshita^1†^, Peter K. Davidsen^1†^, John M. Herbert^1^, Philipp Antczak^1,8^, Matthijs K. C. Hesselink^2^, Patrick Schrauwen^2^, S. John Weisnagel^3^, Jeremy M. Robbins^4^, Robert E. Gerszten^4^, Sujoy Ghosh^5^, Mark A. Sarzynski^6^, Claude Bouchard^7^ and Francesco Falciani^1*^

^1^ Institute of Systems, Molecular and Integrative Biology, Crown Street, Liverpool, L69 7ZB, UK.

^2^ Department of Nutrition and Movement Sciences, NUTRIM School for Nutrition and Translational Research in Metabolism, Maastricht University Centre, Maastricht, The Netherlands.

^3^ Diabetes Research Unit, Endocrinology and Nephrology Axis, CRCHU de Québec, Université Laval, Québec City, Canada.

^4^ Division of Cardiovascular Medicine, and Cardiovascular Research Center, Beth Israel Deaconess Medical Center, Boston, MA 02215, USA

^5^ Centre for Computational Biology and Program in Cardiovascular and Metabolic Disorders, Duke-NUS Medical School, Singapore.

^6^ Department of Exercise Science, Arnold School of Public Health, University of South Carolina, Columbia, SC, USA

^7^ Human Genomics Laboratory, Pennington Biomedical Research Center, Baton Rouge, LA, USA.

^8^ Center for Molecular Medicine Cologne, University Hospital Cologne, 50931, Germany.

* Corresponding Author

† The authors contributed equally to the paper

# Supplementary Methods

**Consent protocols and research integrity**

The study protocol was approved by the Institutional Review Boards at each of the five participating centers of the HERITAGE Family Study consortium (Indiana University, Laval University, University of Minnesota, Texas A&M University, and Washington University at St. Louis). Written informed consent was obtained from each study participant. The subjects who were under 18 years, one of the parents gave consent in addition to the participant. This was an easy procedure to follow as we were recruiting whole nuclear families. All research was performed in accordance with the Declaration of Helsinki.

# Exercise training protocol.

The exercise intensity of the 20-week training program was customized for each participant based on the heart rate-VO2 relationship measured at baseline [1]. During the first two weeks the subjects exercised at a heart rate corresponding to 55% of the baseline VO2max for 30 min per session. Duration and intensity of the sessions were gradually increased to 50 min and 75% of the heart rate associated with baseline VO2max, which were then sustained for the last six weeks of the training.

# Measurements of maximal oxygen uptake (VO_2_max).

Two maximal exercise tests were performed on 2 separate days at baseline and again on 2 separate days after training. A SensorMedics 800S (Yorba Linda, CA) cycle ergometer and SensorMedics 2900 metabolic measurement cart were used. The tests were conducted at about the same time of day, with at least 48 hours between the 2 tests, as described earlier [2]. The mean VO_2_max from the 2 tests was taken as the true max for a given subject and used in analyses if the values were within 5% of each other. If they differed by more than 5%, the higher VO_2_max value was used.

# Venipuncture.

Blood samples were obtained from an antecubital vein into Vacutainer tubes containing EDTA in the morning after a 12-hour fast with participants in a semi-recumbent position. The blood samples were collected twice at baseline (on separate days), and again at 24- and 72-hours after the last training session. For eumenorrheic women, all samples were obtained in the early follicular phase of the menstrual cycle when blood plasma cholesterol alterations are minimal.

# IVGTT protocol.

Fasting plasma insulin and glucose were obtained at baseline and 1 day after the last exercise bout. An intravenous glucose tolerance test (IVGTT) was performed after an overnight fast at baseline, and the post-exercise program IVGTT was performed in the fasted state 1 day after an exercise session. In premenopausal women, the test was scheduled to coincide with the follicular phase of the menstrual cycle. The protocol previously defined by Walton et al. was followed for the IVGTT [3]. The protocol did not include an injection of insulin or tolbutamide.

Plasma insulin was measured by radioimmunoassay after polyethylene glycol separation [4]. Polyclonal antibodies cross-react more than 90% with proinsulin [5]. Therefore, in this study, insulin refers to immunoreactive insulin. In the present cohort with normal fasting glucose levels and no history of diabetes, it is estimated that about 10% of the immunoreactive insulin is in the form of proinsulin and its conversion intermediates [6]. The intra- and inter-assay coefficients of variation were 7.7% and 10.3%, respectively. Plasma glucose was enzymatically determined using a reagent kit (Diagnostic Chemicals Ltd).

From the MINMOD Millennium software [7], we derived the following traits: S_i_, AIR_g_, D_I_, and S_g_. S_i_, the insulin sensitivity index, measures the ability of an increment in plasma insulin to enhance the net disappearance of glucose from plasma and is used as a measure of insulin sensitivity. AIR_g_, acute insulin response to glucose, is defined as the integrated area under the insulin curve between 0 and 40 minutes of the IVGTT and represents a measure of insulin response. D_I_, disposition index, was calculated as S_i_ multiplied by AIR_g_ and measures the ability of the pancreatic beta cells to compensate for changes in insulin sensitivity. S_g_, glucose effectiveness, measures the ability of glucose per se, independently of change in plasma insulin, to increase glucose disposal and to suppress endogenous glucose output.

**Descriptive statistics of healthy individuals enrolled in the HERITAGE study**

|  | Subjects with GWAS data | | | | Subsample with gene expression data | | | |
| --- | --- | --- | --- | --- | --- | --- | --- | --- |
| **Variable** | **N** | **Mean (SD)** | **95% CI** | **Range** | **N** | **Mean (SD)** | **95% CI** | **Range** |
| **Age** | 479 | 35.89 (14.58) | 34.59 to 37.2 | 17.02 to 65.21 | 41 | 34.28 (14.49) | 29.84 to 38.72 | 17.36 to 62.5 |
| **Gender** |  |  |  |  |  |  |  |  |
| *Male* | 233 | - | - | - | 24 | - | - | - |
| *Female* | 246 | - | - | - | 17 | - | - | - |
| **BMI**, kg/m^2^ |  |  |  |  |  |  |  |  |
| *Baseline* | 479 | 25.84 (4.97) | 25.4 to 26.29 | 17.03 to 47.54 | 41 | 26.03 (3.89) | 24.84 to 27.23 | 20.63 to 36.58 |
| *Response to Training* | 477 | -0.09 (0.75) | -0.16 to -0.02 | -3.5 to 2.87 | 41 | 0.07 (0.77) | -0.16 to 0.31 | -1.34 to 2.11 |
| **Plasma Glucose**, mmol/L |  |  |  |  |  |  |  |  |
| *Baseline* | 463 | 5.1 (0.93) | 5.01 to 5.18 | 2.8 to 13 | 41 | 5.63 (1.14) | 5.28 to 5.98 | 4 to 9.3 |
| *Response to Training* | 443 | 0.15 (1.17) | 0.05 to 0.26 | -4.4 to 5.1 | 40 | 0.09 (1.32) | -0.32 to 0.5 | -2.7 to 3.3 |
| **Plasma Insulin**, pmol/L |  |  |  |  |  |  |  |  |
| *Baseline* | 468 | 65.75 (40.04) | 62.12 to 69.38 | 9 to 396 | 41 | 61.44 (34.06) | 51.01 to 71.86 | 15 to 160 |
| *Response to Training* | 455 | -5.16 (24.97) | -7.46 to -2.87 | -183 to 82 | 41 | -2.54 (19.33) | -8.45 to 3.38 | -59 to 40 |
| **S_I_**, mU / [L x min] |  |  |  |  |  |  |  |  |
| *Baseline* | 451 | 4.35 (2.94) | 4.08 to 4.62 | 0.03 to 19.6 | 40 | 5.42 (4.12) | 4.14 to 6.7 | 0.03 to 19.6 |
| *Response to Training* | 429 | 0.25 (2.69) | 0 to 0.51 | -15.4 to 11 | 39 | -1 (3.75) | -2.17 to 0.18 | -15.4 to 3.14 |
| **VO2max**, mL O_2_ / (kg x min) |  |  |  |  |  |  |  |  |
| *Baseline* | 469 | 33.16 (8.86) | 32.36 to 33.96 | 14.92 to 57.03 | 41 | 35.72 (9.04) | 32.95 to 38.49 | 18.61 to 57.03 |
| *Response to Training* | 467 | 5.45 (3.03) | 5.17 to 5.72 | -2.19 to 15.93 | 41 | 5.08 (2.52) | 4.31 to 5.85 | 1.07 to 12.09 |
| **Type I Fibre (%)** |  |  |  |  |  |  |  |  |
| *Baseline* | - | - | - | - | 41 | 44.71 (11.58) | 41.16 to 48.25 | 18.4 to 70.4 |
| *Response to Training* | - | - | - | - | 41 | 2.14 (9.71) | -0.83 to 5.11 | -14.9 to 28.9 |

# Supplementary Results

We hypothesised that MEF2A could be a key driver of the pleiotropic transcriptional response linked to ΔS_i_. In order to validate the prediction of our analysis, we first defined the global transcriptional signature associated to MEF2A knockdown and then asked whether this experimentally determined MEF2A-dependent signature can recapitulate the transcriptional signature correlated to ΔS_i_ in the HERITAGE cohort. Recently, Wales et al. [8] reported a comprehensive list of both direct and indirect MEF2A mammalian target genes using isoform-specific short interference RNA (siRNA) knockdown in differentiating C2C12 myotubes. Notably, despite the four-membered MEF2 protein family sharing a high sequence homology in their DNA binding domains, genes distinctly sensitive to the A isoform play roles in calcium signalling [9].

Of the 1,280 dysregulated genes reported by Wales et al. (828 being downregulated), we identified human orthologs for 990 (77%; **Supplementary** **Table S4**). We then performed a custom GSEA using up- and down-regulated MEF2A gene targets, respectively, as gene sets. Intriguingly, genes downregulated by knockdown of MEF2A, which overall relate to ‘muscle function’ [8], were highly enriched (FDR<1%) amongst the most positively associated genes to ΔS_i_ in HERITAGE (**Figure 1**). In addition, the second gene set containing upregulated MEF2A target genes was highly enriched (FDR<1%) amongst the most negatively associated genes to ΔS_i_.


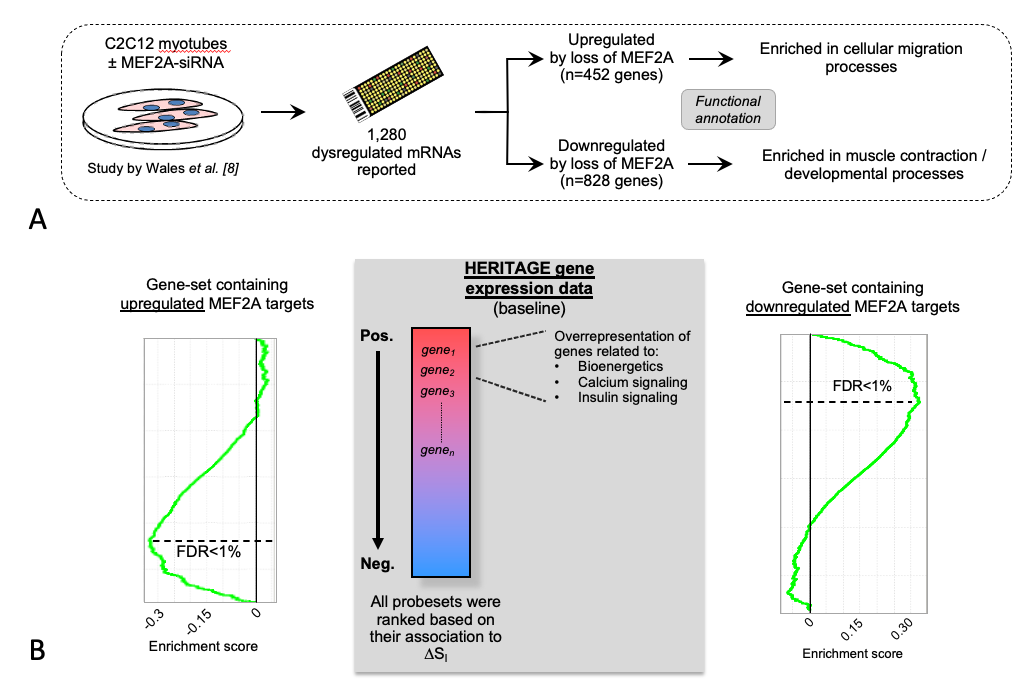


**Figure 1.** (A) Using small interfering RNA (siRNA), Wales *et al.* [8] recently defined the global transcriptional signature associated with MEF2A modulation in differentiating C2C12 myotubes. Corresponding human orthologs for the dysregulated genes were identified using the Mouse Genome Informatics (MGI) database (72% mapping success). (B) All skeletal muscle expressed genes in HERITAGE were ranked according to their association to ∆S_I_ (grey panel in the middle). GSEA revealed that the custom gene set containing gene targets upregulated by MEF2A knockdown was enriched in the negative end of the ranked gene list (left panel, core enriched genes = 91), whereas the gene set containing downregulated MEF2A targets was enriched in the positive end (right panel, core enriched genes = 146). The green curves correspond to the running sum of the enrichment score that reflects the degree to which the MEF2A-associated signatures are overrepresented at the top or bottom of the ranked list.

# Supplementary Discussion

# Here we expand the discussion on genes identified via GWAS analysis which are not explored in detail in the main manuscript. These genes were either not present in the IMPC database and no information on their knockout in mice was available, or they were present in the database but traits relevant to S_I_ were not significantly changed with the gene knockout.

Regarding baseline S_I_ associated genes, rs11622678 (positionally mapped to *DNAL1*) is also an eQTL to *PNMA1* in skeletal muscle, according to GTEx database [10]. PNMA1 has been associated to cell proliferation as an apoptosis regulator, and it has been shown to be oncogenic in pancreatic cancer via activation of PI3K/AKT and MAP/ERK pathways, and in hepatocellular carcinoma by activating Wnt/Beta-catenin pathway [11]. It has been found downregulated in peripheral blood mononuclear cells of obese compared to lean individuals [12]. *PRKACB* (positionally mapped to rs7524898) is involved in cell signalling by encoding a protein that activates protein kinase A (PKA) [13]. Several biological processes are affected by PKA activation, some of them being lipolysis in adipocytes, insulin secretion and insulin signalling [14]. *CHODL* (positionally mapped to rs205666) has been suggested to play a role in motor axon growth in neuromuscular junction [15–17], potentially being involved in muscle development. However, no mutations in these gene have been previously associated to S_I_ or other related phenotypes.

Regarding ΔS_I_ candidate genes, *SPATA16* has been mainly associated to male infertility since it is involved in spermatogenesis [18] and knockout of this gene in mice leads to both male and female infertility [19]. *WWC1*, mapped to an intronic SNP, encodes KIBRA protein which is mostly expressed in kidney and brain and has been linked to human memory. It has been mapped to GWAS loci associated with BMI in Caucasians [20, 21] and with diabetic nephropathy in an Asian population [22], and it has been found downregulated in differentiated myotubes compared to undifferentiated [23]. *FAM228A* (identified via eQTL mapping) have unknown function, but this gene was mapped to a genetic variant identified in a GWAS investigating genetic variability influencing differences in muscle mass [24]. The *CDH13* gene, mapped to an intronic SNP, encodes for T-cadherin, which has been implicated in several biological processes. Most cadherins are involved in cell adhesion, but this atypical cadherin lacks a transmembrane and cytoplasmic domain, being anchored to the membrane through glycosylphosphatidylinositol [25]. Among other molecules T-cadherin is a receptor for adiponectin, which is a hormone secreted by adipocytes that targets skeletal muscle cells promoting insulin sensitivity, fatty-acid oxidation, and anti-atherosclerotic effects. It has also been shown to exert anti-inflammatory, oxidative and pro-myogenic effects on skeletal muscle exposed to acute or chronic inflammation and injury via T-cadherin [26]. Several GWAS identified SNPs associated to adiponectin levels mapping to *CDH13* [27–33]. Other relevant traits associated to markers mapped to this gene include BMI, waist-hip ratio, glucose homeostasis and levels of trans fatty acids [20, 21, 34–36].

# References

1. Bouchard C, Rankinen T, Chagnon YC, Rice T, Pérusse L, Gagnon J, et al. Genomic scan for maximal oxygen uptake and its response to training in the HERITAGE Family Study. Journal of applied physiology (Bethesda, Md : 1985). 2000;88:551–9.

2. Skinner JS, Wilmore KM, Jaskolska A, Jaskolski A, Daw EW, Rice T, et al. Reproducibility of maximal exercise test data in the HERITAGE family study. Medicine and science in sports and exercise. 1999;31:1623–8.

3. Walton C, Godsland IF, Proudler AJ, Felton C, Wynn V. Evaluation of four mathematical models of glucose and insulin dynamics with analysis of effects of age and obesity. The American journal of physiology. 1992;262 5 Pt 1:E755-62.

4. Desbuquois B, Aurbach GD. Use of polyethylene glycol to separate free and antibody-bound peptide hormones in radioimmunoassays. The Journal of clinical endocrinology and metabolism. 1971;33:732–8.

5. Røder ME, Porte D, Schwartz RS, Kahn SE. Disproportionately elevated proinsulin levels reflect the degree of impaired B cell secretory capacity in patients with noninsulin-dependent diabetes mellitus. The Journal of clinical endocrinology and metabolism. 1998;83:604–8.

6. Kahn SE, Leonetti DL, Prigeon RL, Boyko EJ, Bergstrom RW, Fujimoto WY. Relationship of proinsulin and insulin with noninsulin-dependent diabetes mellitus and coronary heart disease in Japanese-American men: impact of obesity--clinical research center study. The Journal of clinical endocrinology and metabolism. 1995;80:1399–406.

7. Boston RC, Stefanovski D, Moate PJ, Sumner AE, Watanabe RM, Bergman RN. MINMOD Millennium: a computer program to calculate glucose effectiveness and insulin sensitivity from the frequently sampled intravenous glucose tolerance test. Diabetes technology & therapeutics. 2003;5:1003–15.

8. Wales S, Hashemi S, Blais A, McDermott JC. Global MEF2 target gene analysis in cardiac and skeletal muscle reveals novel regulation of DUSP6 by p38MAPK-MEF2 signaling. Nucleic Acids Res. 2014;42:11349–62.

9. Estrella NL, Desjardins CA, Nocco SE, Clark AL, Maksimenko Y, Naya FJ. MEF2 transcription factors regulate distinct gene programs in mammalian skeletal muscle differentiation. J Biol Chem. 2015;290:1256–68.

10. GTEx Consortium, Laboratory, Data Analysis &Coordinating Center (LDACC)—Analysis Working Group, Statistical Methods groups—Analysis Working Group, Enhancing GTEx (eGTEx) groups, NIH Common Fund, NIH/NCI, et al. Genetic effects on gene expression across human tissues. Nature. 2017;550:204–13.

11. Liu P, Chen B, Gu Y, Liu Q. PNMA1, regulated by miR-33a-5p, promotes proliferation and EMT in hepatocellular carcinoma by activating the Wnt/β-catenin pathway. Biomedicine & Pharmacotherapy. 2018;108:492–9.

12. Abu-Farha M, Tiss A, Abubaker J, Khadir A, Al-Ghimlas F, Al-Khairi I, et al. Proteomics Analysis of Human Obesity Reveals the Epigenetic Factor HDAC4 as a Potential Target for Obesity. PLoS One. 2013;8. doi:10.1371/journal.pone.0075342.

13. Hofmann B, Nishanian P, Nguyen T, Insixiengmay P, Fahey JL. Human immunodeficiency virus proteins induce the inhibitory cAMP/protein kinase A pathway in normal lymphocytes. Proc Natl Acad Sci U S A. 1993;90:6676–80.

14. Kanehisa M, Goto S. KEGG: kyoto encyclopedia of genes and genomes. Nucleic Acids Res. 2000;28:27–30.

15. Oprişoreanu A-M, Smith HL, Arya S, Webster R, Zhong Z, Wehner D, et al. Interaction of Axonal Chondrolectin with Collagen XIXa1 Is Necessary for Precise Neuromuscular Junction Formation. Cell Rep. 2019;29:1082-1098.e10.

16. Narcís JO, Tapia O, Tarabal O, Piedrafita L, Calderó J, Berciano MT, et al. Accumulation of poly(A) RNA in nuclear granules enriched in Sam68 in motor neurons from the SMNΔ7 mouse model of SMA. Sci Rep. 2018;8:9646.

17. Sleigh JN, Barreiro-Iglesias A, Oliver PL, Biba A, Becker T, Davies KE, et al. Chondrolectin affects cell survival and neuronal outgrowth in in vitro and in vivo models of spinal muscular atrophy. Hum Mol Genet. 2014;23:855–69.

18. Xu M, Xiao J, Chen J, Li J, Yin L, Zhu H, et al. Identification and characterization of a novel human testis-specific Golgi protein, NYD-SP12. Mol Hum Reprod. 2003;9:9–17.

19. Dickinson ME, Flenniken AM, Ji X, Teboul L, Wong MD, White JK, et al. High-throughput discovery of novel developmental phenotypes. Nature. 2016;537:508–14.

20. Kichaev G, Bhatia G, Loh P-R, Gazal S, Burch K, Freund MK, et al. Leveraging Polygenic Functional Enrichment to Improve GWAS Power. Am J Hum Genet. 2019;104:65–75.

21. Zhu Z, Guo Y, Shi H, Liu C-L, Panganiban RA, Chung W, et al. Shared genetic and experimental links between obesity-related traits and asthma subtypes in UK Biobank. J Allergy Clin Immunol. 2020;145:537–49.

22. Taira M, Imamura M, Takahashi A, Kamatani Y, Yamauchi T, Araki S-I, et al. A variant within the FTO confers susceptibility to diabetic nephropathy in Japanese patients with type 2 diabetes. PLoS ONE. 2018;13:e0208654.

23. Tomczak KK, Marinescu VD, Ramoni MF, Sanoudou D, Montanaro F, Han M, et al. Expression profiling and identification of novel genes involved in myogenic differentiation. FASEB J. 2004;18:403–5.

24. Hernandez Cordero AI, Gonzales NM, Parker CC, Sokolof G, Vandenbergh DJ, Cheng R, et al. Genome-wide Associations Reveal Human-Mouse Genetic Convergence and Modifiers of Myogenesis, CPNE1 and STC2. Am J Hum Genet. 2019;105:1222–36.

25. Philippova M, Joshi MB, Kyriakakis E, Pfaff D, Erne P, Resink TJ. A guide and guard: The many faces of T-cadherin. Cellular Signalling. 2009;21:1035–44.

26. Abou-Samra M, Selvais CM, Dubuisson N, Brichard SM. Adiponectin and Its Mimics on Skeletal Muscle: Insulin Sensitizers, Fat Burners, Exercise Mimickers, Muscling Pills … or Everything Together? International Journal of Molecular Sciences. 2020;21:2620.

27. Wu Y, Gao H, Li H, Tabara Y, Nakatochi M, Chiu Y-F, et al. A meta-analysis of genome-wide association studies for adiponectin levels in East Asians identifies a novel locus near WDR11-FGFR2. Hum Mol Genet. 2014;23:1108–19.

28. Aslibekyan S, An P, Frazier-Wood AC, Kabagambe EK, Irvin MR, Straka RJ, et al. Preliminary evidence of genetic determinants of adiponectin response to fenofibrate in the Genetics of Lipid Lowering Drugs and Diet Network. Nutr Metab Cardiovasc Dis. 2013;23:987–94.

29. Dastani Z, Hivert M-F, Timpson N, Perry JRB, Yuan X, Scott RA, et al. Novel loci for adiponectin levels and their influence on type 2 diabetes and metabolic traits: a multi-ethnic meta-analysis of 45,891 individuals. PLoS Genet. 2012;8:e1002607.

30. Morisaki H, Yamanaka I, Iwai N, Miyamoto Y, Kokubo Y, Okamura T, et al. CDH13 gene coding T-cadherin influences variations in plasma adiponectin levels in the Japanese population. Hum Mutat. 2012;33:402–10.

31. Chung C-M, Lin T-H, Chen J-W, Leu H-B, Yang H-C, Ho H-Y, et al. A genome-wide association study reveals a quantitative trait locus of adiponectin on CDH13 that predicts cardiometabolic outcomes. Diabetes. 2011;60:2417–23.

32. Jee SH, Sull JW, Lee J-E, Shin C, Park J, Kimm H, et al. Adiponectin concentrations: a genome-wide association study. Am J Hum Genet. 2010;87:545–52.

33. Wu Y, Li Y, Lange EM, Croteau-Chonka DC, Kuzawa CW, McDade TW, et al. Genome-wide association study for adiponectin levels in Filipino women identifies CDH13 and a novel uncommon haplotype at KNG1-ADIPOQ. Hum Mol Genet. 2010;19:4955–64.

34. Pulit SL, Stoneman C, Morris AP, Wood AR, Glastonbury CA, Tyrrell J, et al. Meta-analysis of genome-wide association studies for body fat distribution in 694 649 individuals of European ancestry. Hum Mol Genet. 2019;28:166–74.

35. Palmer ND, Goodarzi MO, Langefeld CD, Wang N, Guo X, Taylor KD, et al. Genetic Variants Associated With Quantitative Glucose Homeostasis Traits Translate to Type 2 Diabetes in Mexican Americans: The GUARDIAN (Genetics Underlying Diabetes in Hispanics) Consortium. Diabetes. 2015;64:1853–66.

36. Mozaffarian D, Kabagambe EK, Johnson CO, Lemaitre RN, Manichaikul A, Sun Q, et al. Genetic loci associated with circulating phospholipid trans fatty acids: a meta-analysis of genome-wide association studies from the CHARGE Consortium. Am J Clin Nutr. 2015;101:398–406.
